# Impact of automated ICA-based denoising of fMRI data in acute stroke patients

**DOI:** 10.1016/j.nicl.2017.06.033

**Published:** 2017-06-30

**Authors:** D. Carone, R. Licenik, S. Suri, L. Griffanti, N. Filippini, J. Kennedy

**Affiliations:** a Acute Vascular Imaging Centre, Radcliffe Department of Medicine, University of Oxford, Oxford, United Kingdom; b Laboratory of Experimental Stroke Research, Department of Surgery and Translational Medicine, University of Milano Bicocca, Milan Center of Neuroscience, Monza, Italy; c Department of Social Medicine and Public Health, Faculty of Medicine, Palacky University, Olomouc, Czech Republic; d Department of Psychiatry, Warneford Hospital, University of Oxford, Oxford, United Kingdom; e Oxford Centre of Functional MRI of the Brain, Nuffield Department of Clinical Neurosciences, University of Oxford, Oxford, United Kingdom; f Nuffield Department of Clinical Neurosciences, West Wing level 6, JR hospital, Oxford, United Kingdom

**Keywords:** Independent component analysis, fMRI, Acute stroke, Denoising, BOLD, Resting state

## Abstract

Different strategies have been developed using Independent Component Analysis (ICA) to automatically de-noise fMRI data, either focusing on removing only certain components (e.g. motion-ICA-AROMA, Pruim et al., 2015a) or using more complex classifiers to remove multiple types of noise components (e.g. FIX, Salimi-Khorshidi et al., 2014 Griffanti et al., 2014). However, denoising data obtained in an acute setting might prove challenging: the presence of multiple noise sources may not allow focused strategies to clean the data enough and the heterogeneity in the data may be so great to critically undermine complex approaches. The purpose of this study was to explore what automated ICA based approach would better cope with these limitations when cleaning fMRI data obtained from acute stroke patients.

The performance of a focused classifier (ICA-AROMA) and a complex classifier (FIX) approaches were compared using data obtained from twenty consecutive acute lacunar stroke patients using metrics determining RSN identification, RSN reproducibility, changes in the BOLD variance, differences in the estimation of functional connectivity and loss of temporal degrees of freedom.

The use of generic-trained FIX resulted in misclassification of components and significant loss of signal (< 80%), and was not explored further. Both ICA-AROMA and patient-trained FIX based denoising approaches resulted in significantly improved RSN reproducibility (*p* < 0.001), localized reduction in BOLD variance consistent with noise removal, and significant changes in functional connectivity (*p* < 0.001). Patient-trained FIX resulted in higher RSN identifiability (*p* < 0.001) and wider changes both in the BOLD variance and in functional connectivity compared to ICA-AROMA.

The success of ICA-AROMA suggests that by focusing on selected components the full automation can deliver meaningful data for analysis even in population with multiple sources of noise. However, the time invested to train FIX with appropriate patient data proved valuable, particularly in improving the signal-to-noise ratio.

## Highlights

• Two ICA based de-noising tools (FIX and AROMA) are tested on acute stroke fMRI data.
• Both FIX and AROMA significantly increased RSNs reproducibility.
• Their use led to changes in functional connectivity estimates and BOLD signal variance.
• FIX in order to be accurate required to be trained on appropriate patient data.
• FIX in contrast to AROMA additionally increased RSNs identifiability.

## 1 Introduction

After acute brain injury, brain plasticity and reorganization underlie functional recovery, and improvement is mediated by adaptations in brain network connectivity (Andrews, 1991). Resting-state functional MRI (rs-fMRI) allows detailed investigation of these changes providing potentially useful information to guide treatment. The minimal demands on the patient during scanning makes the technique an ideal and increasingly used choice in acute clinical settings (Ovadia-Caro et al., 2014).

However, overcoming the contribution of noise in the acquisition of rs-fMRI data is important in limiting false observations and reliably estimating functional connectivity in Resting State Networks (RSN) (Raichle and Snyder, 2007, Friston et al., 1996, Dagli et al., 1999, Glover et al., 2000, Windischberger et al., 2002). Independent component analysis (ICA) can be used to reliably separate signal from noise, enabling artefacts to be regressed out of the data thereby allowing a significant improvement on results obtained with traditional pre-processing (Stone et al., 2002, Thomas et al., 2002, Kochiyama et al., 2005, McKeown et al., 2005, Zou et al., 2009, Zuo and Xing, 2014). While manual component classification has been widely used as the gold standard (De Martino et al., 2007, Bhaganagarapu et al., 2013, Rummel et al., 2013, Salimi-Khorshidi et al., 2014), it is time-consuming, operator dependent and requires expert knowledge to separate signal and noise characteristics.

There have been a variety of attempts to automate the classification of independent components (ICs) (Thomas et al., 2002, Kochiyama et al., 2005, De Martino et al., 2007, Perlbarg et al., 2007, Tohka et al., 2008, Kundu et al., 2012, Bhaganagarapu et al., 2013, Rummel et al., 2013, Storti et al., 2013, Salimi-Khorshidi et al., 2014, Pruim et al., 2015a). Some avoid training altogether, opting for a fully automated and robustly generalizable approach by making it specific to certain components (Caballero-Gaudes and Reynolds, 2016). An example of such an approach would be ICA-based Automatic Removal of Motion Artefacts (ICA-AROMA), which focuses on removing only components associated with motion (Pruim et al., 2015a). A more complex approach, such as FMRIB's ICA-based X-noiseifier (FIX) (Salimi-Khorshidi et al., 2014, Griffanti et al., 2014), confers the theoretical opportunity of removing more sources of noise thereby improving the accuracy of any signal changes identified. This comes at the cost of reduced generalizability, requiring re-training for different datasets and the possibility of limited accuracy when applied to heterogeneous data (Salimi-Khorshidi et al., 2014, Pruim et al., 2015b).

The limitations of either approach to noise removal are potentially more apparent when applied in acute clinical populations, where multiple sources of noise may be simultaneously more evident and heterogeneous than in cohorts that are either healthy, or have a chronic disease process such as dementia. An acute stroke population is a good example of this; up to 35% of stroke patients present with aphasia (Pedersen et al., 1995), which may impact their compliance with instruction in the MRI scanner with a likely increase in motion artefact. Noise secondary to modifications of cardiac/respiratory cycles (Birn et al., 2006, Shmueli et al., 2007) are likely to be more prevalent given how common both cardiac arrhythmias (for example, atrial fibrillation), and alterations in breathing control, respiratory mechanics, and breathing pattern are in this population (Engstrom et al., 2000, Nogues and Benarroch, 2008). Artefact due to altered vascular tone is also likely to be present following recruitment of collateral circulation (Liebeskind, 2003) and disruption of cerebrovascular regulatory mechanisms (Kunz and Iadecola, 2009) in response to cerebral ischemia.

Limiting noise removal to a single aspect, such as motion, may not clean the data enough to enable meaningful analysis. The classification accuracy of a complex approach could diminish if the characteristics of the data to analyse deviate substantially from the training data, particularly in the presence of different scanners or acquisition protocols (Caballero-Gaudes and Reynolds, 2016, Pruim et al., 2015b, Salimi-Khorshidi et al., 2014).

In addition, grouping acute stroke patients for analysis will inevitably add further variability due to differing clinical presentation, lesion localization, haemodynamic parameters, and vasoactive medications all of which will impact the interpretation of signal changes. These wide and inconsistent potential sources of variance might undermine the clinical applicability of a complex approach to IC classification.

The purpose of this study was to explore how these limitations are addressed in using ICA denoising approaches applied to a dataset obtained from a cohort of acute stroke patients. The performance of a focused classifier approach (ICA-AROMA) and complex classifier (FIX) were compared using metrics determining RSN identification, RSN reproducibility, changes in the BOLD variance and differences in the estimation of functional connectivity.

## 2 Methods

### 2.1 Subjects and MRI data acquisition

Consecutive patients presenting with a clinical lacunar stroke syndrome within 24 h of symptom onset (using the last seen well principle) regardless of age or stroke severity were recruited into a prospective observational cohort study following informed consent or agreement from a representative according to protocols approved by UK National Research Ethics Service committees (ref: 12/SC/0292 and 13/SC/0362). Exclusion criteria included the presence of a contraindication for MRI. All clinical decisions were made prior to enrolment in order not to introduce any delay to best clinical care.

Data were acquired using a Siemens 3T Verio scanner (Siemens Healthcare, Erlangen, Germany) with a 32-channel head coil. Whenever possible, each subject was scanned at presentation, 24 h, a week and a month later. At each time point, a resting state T2*-weighted scan (gradient echo EPI with TR = 3000 ms, TE = 40 ms, flip angle = 90; voxel size: 3.0 × 3.0 × 3.0 mm, 120 frames, 6 min 6 s) and a T1-weighted MP-RAGE (1.8 × 1.8 × 1.0 mm, field of view = 228 mm, TR = 2040 ms, TE = 4.55 ms, TI = 900 ms, 3 min 58 s) were acquired.

### 2.2 Resting state fMRI data pre-processing and single subject IC analysis

Prior to the use of either ICA-AROMA or FIX, rs-fMRI data were pre-processed using EPI distortion correction, rigid-body registration, brain extraction, Gaussian smoothing with a 5 mm FWHM kernel. Each 4D pre-processed dataset entered single-subject spatial-ICA decomposition with automatic dimensionality estimation using Multivariate Exploratory Linear Optimised Decomposition of Independent Components (MELODIC) (Beckmann and Smith, 2004). High-pass temporal filtering (cut off = 100 s) was also applied to the rs-fMRI data before ICA when using FIX, and, after ICA when using ICA-AROMA in accordance with the respective manuals.

### 2.3 IC classification and noise removal

Independent components were classified into signal or noise both manually and using ICA-AROMA and FIX.

#### 2.3.1 Manual classification

Manual classification was performed using Melview (FSL 5.0, Oxford, UK; www.fmrib.ox.ac.uk/fsl), a visualization software specifically written for FIX to display each IC's spatial map, temporal power spectrum, and time course (default starting threshold used 2). Two independent raters (DC and RL) manually classified the ICs following a standardized procedure (Kelly et al., 2010). Disagreements were discussed with the group and resolved by reference to a third author (NF). The decision to label an IC as noise or signal was based, primarily, upon visual inspection of the spatial map and, secondarily, upon the temporal power spectrum and the time course. ICs were labelled as noise when the cluster peaks were localized predominantly in peripheral areas, or in a spotty or speckled pattern seemingly scattered at random over a large section (roughly 1/4 or more) of the brain without regard for functional–anatomical boundaries. Components were labelled as signal when presenting small to larger clusters localized in small regions of the brain. Additional secondary criteria applied to identify noise were: a predominantly high-frequency spectrum; the presence of spikes or saw-tooth pattern in the time course; the presence of signal in the venous sinuses. Both raters were trained to classify ICs using a dataset derived from five stroke patients not included in the FIX classifier performance analysis (these five patients were included in all other analysis). Training was deemed successful when the inter-rater agreement ratio for IC classification in the dummy dataset reached 95%. Each rater classified the finalized dataset twice, separated by one month. Inter-rater agreement and intra-rater agreements were calculated.

#### 2.3.2 Automatic classification

Automatic classification using FIX was performed using 2 different training procedures:

• Generic training, using the standard training-weight file supplied with FIX (Salimi-Khorshidi et al., 2014), created from a cohort of healthy volunteers (Standard EPI acquisition, single protocol, on a Siemens 3T Verio, voxel size = 3 × 3 × 3 mm^3^, TR = 3000 ms).
• Patient (study-specific) training using the hand labels obtained from manually classifying the fMRI data acquired from the study patients.

ICs that where classified as noise were subsequently regressed out from the rs-fMRI data using the non-aggressive option, ensuring that only the unique artefact-related variance was removed (Griffanti et al., 2014).

#### 2.3.3 ICA-AROMA

ICA-AROMA was applied after spatial smoothing and before high-pass temporal filtering. ICs that were classified as motion-related were regressed out of the data following the non-aggressive option (Pruim et al., 2015a).

### 2.4 FIX classification performances indices

FIX classifier performance was summarised by the ability to identify signal and noise components when compared to the agreed manual classification. Two measures were calculated: true positive ratio (TPR, the percentage of true signal components correctly detected) and true negative ratio (TNR, the percentage of true artefact components correctly detected).

FIX performance following generic and patient training on the patient data, was tested using a leave-one-subject-out (LOSO) approach across sets of ICA output components to avoid overfitting. If the training data consists of n datasets, each fold of the cross validation uses n-1 datasets for training, and tests the learned decision boundary of the left-out dataset. In the case of having multiple runs of data from each patient, it was considered safest to leave out all runs for a given patient and train on all datasets of all other patient in order to generalise LOSO accuracy results when applied to future patients.

A threshold is applied to FIX output to determine the binary classification of any given component. Changing the threshold shifts the balance between TPR and TNR. Several thresholds were evaluated in order to show how to find the ideal balance between prioritizing TNR against TPR (Salimi-Khorshidi et al., 2014). The optimal threshold was defined as the one that would grant the highest TNR (at least 10%) while preserving a TPR above 90%.

An overlap analysis was performed (number of voxel overlapping normalized by the total number of voxels of the components) to explore a possible link between the ischemic lesion localization and mislabelled components.

### 2.5 Estimating functional connectivity from template-based dual regression

After pre-processing, the functional scans were registered to the MNI152 standard space (Montreal Neurological Institute, Montreal, QC, Canada) via a transformation matrix obtained from aligning the subject's functional scan to the anatomical scan, and the anatomical scan to the MNI152 template (12 DOF). Functional connectivity analysis was run both on corrected data and on original (pre-processed, uncorrected) data using a template-based dual regression (Beckmann et al., 2009, Filippini et al., 2009) to allow direct comparison among the results obtained. Functional connectivity was defined by fitting the BOLD fluctuations at each voxel with respect to the dominant fluctuation within twenty standardized networks of interest (NOIs) (Smith et al., 2009). This involved dual regression analysis estimating the temporal profile of BOLD variations within each weighted NOI map, followed by using those temporal profiles as regressors to estimate the fit between BOLD fluctuations across the brain and that given NOI. This yields functional connectivity maps in terms of *Z*-scores of the fit that may be used later in group-level analyses. The distribution of the Z-scores across voxels was assessed for every map obtained. Representative histograms are included in the Supplementary Material (Supplementary Material Fig. 1).

In all stages, tools from the Functional Magnetic Resonance Imaging of the Brain (FMRIB) Software Library (FSL 5.0, Oxford, UK; www.fmrib.ox.ac.uk/fsl) were used.

### 2.6 RSN identifiability

RSN identifiability was defined as the ratio between the mean absolute *Z*-score inside and outside of a RSN mask (Pruim et al., 2015b). RSN masks were obtained by thresholding the original templates at | Z | > 2.3. RSN identifiability represents a signal to noise ratio of every participant-level RSN spatial map with a ratio > 1 indicating increased RSN identifiability. This analysis was repeated three further times: firstly, after regressing the artefactual components using the aggressive option; and secondly, using a more lenient threshold (| Z | > 1.5) and, separately, a more conservative threshold (| Z | > 3.1) for the creation of the RSN masks.

### 2.7 RSN reproducibility

RSN reproducibility was investigated using split-half reproducibility (Pruim et al., 2015b). The patient group was randomly divided into two equally sized groups. For both groups, the average group-level spatial parameter estimate map across patients was derived for each of the twenty NOI as estimated by dual regression. The group-level maps were masked to only include gray matter voxels using a MNI152 gray matter probability map thresholded at 50%. Subsequently the between-group spatial correlation for each spatial map was determined yielding a 20 × 20 correlation matrix. The correlation values on the diagonal of this matrix expressed the reproducibility of each IC between groups. In contrast, the off diagonal correlations expressed the spatial correlation between non-matching IC templates. The off diagonal correlations were used as a null-distribution to convert the spatial correlations for the 20 matching RSNs to pseudo *Z*-scores. The reproducibility analyses were conducted for 500 random group splits to obtain average Z-scores and standard deviations.

### 2.8 Loss in temporal degrees of freedom

The loss in temporal degrees of freedom (tDoF) associated with the different strategies was evaluated to investigate its potential impact on statistical power and between group bias (Pruim et al., 2015b). Loss of tDoF was determined for every patient for each strategy by considering every component that was regressed out from the data as a single tDoF. The total available tDoF was defined as the total number of volumes within the fMRI time-series.

### 2.9 Effect of correction on BOLD variance

To test the effect of correction on BOLD variance, a voxel-wise variable, %ΔSTD_map_, was calculated (Khalili-Mahani et al., 2013) for every scan obtained. %ΔSTD_map_ was defined as:

$$\%\Delta {\mathrm{STD}}_{\mathrm{map}}=\left(\mathrm{STD}\;\left({\mathrm{rs}‐\text{fMRI}}_{\text{original}}\right)-\mathrm{STD}\;\left({\mathrm{rs}‐\text{fMRI}}_{\text{corrected}}\right)\right)/\mathrm{STD}\;\left({\mathrm{rs}‐\text{fMRI}}_{\text{original}}\right)\times 100$$

where STD is the standard deviation of each voxel over the acquired volumes of BOLD data. The %ΔSTD maps were then registered to standard space using the affine transformation matrix obtained from registering the rs-fMRI data to MNI152 (using FLIRT, FSL linear transformation tool, FSL 5.0) and used to perform group analysis (one-sample *t*-test) to determine in which regions %ΔSTD was significantly (*p* < 0.01, TFCE) higher than 35%, 45%, 55%, 65%. ΔSTD maps were also thresholded (25%), binarised and averaged to generate a probability map that would highlight in which areas the rs-fMRI variance was more frequently reduced across subjects.

### 2.10 Effect of correction on functional connectivity

The effect of ICA-AROMA and FIX on resting state connectivity was assessed by measuring the percentage of absolute change in the magnitude of Z-scores for each NOI and for each Z-score map (Khalili-Mahani et al., 2013). This percentage change in Z score (|%ΔZ |) was defined as:

$$\left|\%\mathrm{\Delta Z}\right|=|\left(\left(Z_{\text{Corrected}}-{\;Z}_{\text{Original}}\right)/Z_{\text{Original}}\right)\;)\times 100|$$

where Z is obtained summing the values of the voxels of a Z-score map within a NOI template mask.

To determine if there were differences in the effect of using ICA-AROMA or FIX across NOIs, a one-way ANOVA was used with Bonferroni correction for multiple comparisons (values were considered significant if *p* < 0.05). A paired sample *t*-test was used to compare differences between FIX and ICA-AROMA (values were considered significant if *p* < 0.05).

This variable allowed testing whether a significant change in resting-state network consistency results from the use of ICA-AROMA and FIX.

For each denoising approach, spatial maps representing significant changes (*p* < 0.05 TFCE) in functional connectivity at a group level were generated for the 10 “well defined” networks (Pruim et al., 2015b, Smith et al., 2009).

### 2.11 Grayplots

The effect of each denoising procedure was assessed at a single scan level by creating a corresponding grayplot (Power, 2017), a 2-dimensional heatmap of the relevant time series within a scan with time on the x-axis and voxel of region of interest defining the y axis.

## 3 Results

### 3.1 Patients

Twenty patients (age range 56–94; 6 female (35%)) were enrolled consecutively. Onset to first scan time ranged between 2 and 19 h. Patient factors are summarised in Table 1.

**Table 1:** Patients clinical presentation, hemodynamic parameters, medication, brain lesion localization and scans time-points. sO_2_ = oxygen saturation, NIHSS = National Institutes of Health Stroke Scale.

Patient	Age	Sex	Systolic	Diastolic	on H.M.	sO_2_	NIHSS	Hemisphere	Lesion location	Acute scan	24 h scan	Week scan	Month scan
1	65	M	160	70	Y	97	4	R	Posterior limb internal capsule/corona radiata infarct	Y	Y	Y	Y
2	68	F	151	79	Y	94	3	R	Cerebellar infarction	Y	N	N	Y
3	70	M	173	93	N	96	2	L	Posterior limb of internal capsule infarct	Y	N	Y	Y
4	80	M	144	66	Y	100	2	L	Corona radiata infarct	Y	N	N	N
5	93	M	111	65	N	99	4	L	External capsule/lateral putamen/body of the caudate infarct	Y	Y	Y	Y
6	78	M	153	56	N	89	4	R	Multiple foci of cortical/subcortical infarction in the frontal lobe	Y	Y	Y	Y
7	80	M	159	102	N	98	2	R	Corona radiata infarct	Y	Y	Y	Y
8	62	F	148	75	N	100	7	L	Posterior limb of internal capsule/corona radiata infarct	Y	Y	Y	Y
9	71	F	214	74	Y	94	9	R	External capsule/rolandic corona radiata infarct	Y	N	Y	N
10	81	F	145	91	N	96	2	L	External capsule/posterior putamen/corona radiata infarct	Y	N	Y	Y
11	75	F	170	85	Y	96	8	L	Posterior internal capsule/corona radiata infarct	Y	Y	Y	Y
12	72	M	184	110	Y	97	3	R	Head and body caudate and putamen infarct	Y	Y	Y	Y
13	80	M	161	84	Y	98	2	L	Corona radiata/posterior superior insular cortex infarct	Y	Y	N	N
14	55	M	225	173	Y	100	5	L	Corona radiata infarct	Y	Y	Y	N
15	63	M	161	103	N	98	3	L	Several small cortical infarcts	Y	Y	N	N
16	90	M	127	66	Y	97	4	L	Paramedian pontine infarct	N	N	N	Y
17	56	F	145	71	N	95	10	L	Anterior thalamic stroke/cerebral peduncle infarct	Y	N	N	N
18	82	F	166	81	N	95	11	L	Several small cortical infarcts	Y	Y	Y	Y
19	72	M	136	66	N	98	1	L	Posterior part of putamen/corona radiata infarct	Y	Y	Y	Y
20	78	M	112	67	Y	94	1	L	Paramedian pontine infarct	Y	Y	Y	Y

### 3.2 Independent component classification

The average number of single-subject ICs estimated by MELODIC was 53.7 (range 35–68) per scan. The average number of ICs manually classified as signal was 8.2 (range 5–13). The inter-rater agreement ratio was 94.7% (and 94.5% after a month), whilst intra-rater agreement ratio was 95.4% for the first rater and 97.2% for the second rater.

Mean and median values of TPR and TNR using different thresholds are reported in Table 2. When using the generic-training, the requirements for defining an optimal threshold (TPR > 90%) were not met. As a result, data derived from generic-trained FIX were excluded from further analysis. When patient-trained, the optimal threshold for the use of FIX was identified as 20 with a mean (median) TPR and TNR of 91.8% (100%) and 86.9% (88.1%), respectively. The same degree of overlap was found after calculating the overlap between ischemic lesion and correctly labelled components (overlap 0.01), and the overlap between ischemic lesion and misclassified components (overlap 0.01).

**Table 2:** FIX independent component classification performance on the stroke cohort of patients rs-fMRI data, after generic (A) and patient (B) training. TPR = true positive rate, i.e., the percentage of true signals correctly classified. TNR = true negative rate, i.e., the percentage of true artefacts correctly classified. As the FIX threshold is lowered, TPR is maximized at the expense of lower TNR.

A) Generic-trained FIX performance								
Threshold	1	2	5	10	20	30	40	50
Mean TPR	100	100	100	100	76.5	70.5	68.0	53.6
Mean TNR	0	0	0.2	4.0	66.2	78.3	88.1	94.1
Median TPR	100	100	100	100	80	74.6	70.7	55
Median TNR	0	0	0.1	3.2	63.7	78.8	90.4	96.6


B) Patient-trained FIX performance								
Threshold	1	2	5	10	20	30	40	50
Mean TPR	98.1	97.7	97.3	96.3	91.7	88	83.6	79.4
Mean TNR	61.3	65.3	71.8	78.5	86.9	91.6	94	96
Median TPR	100	100	100	100	100	100	88.9	87.5
Median TNR	62.2	66.7	73.6	79.2	88.1	92.3	94.4	96.2

### 3.3 RSN identifiability

**Fig. 1:**
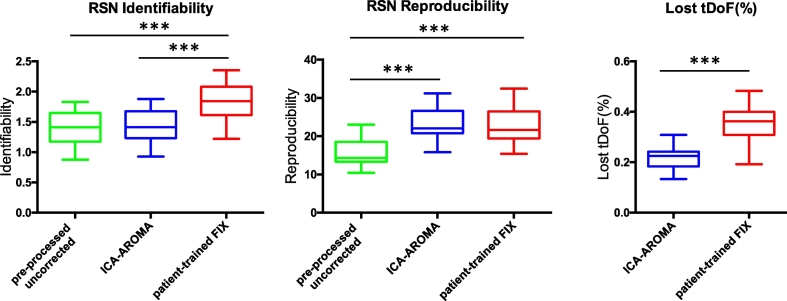
RSN identifiability/reproducibility and lost temporal degrees of freedom (tDoF). RSN identifiability was defined using a Z-score ratio between the Z-scores within and outside a RSN. RSN reproducibility reflects the spatial correlation between group-level RSN maps for random splits of the samples normalized to pseudo Z-scores. The loss of tDoF is expressed as a percentage of the total tDoF, defined as the available number of time points. *** *p* < 0.001.

ICA-AROMA demonstrated RSN identifiability similar to pre-processed uncorrected data (*p* = 0.97). Patient-trained FIX showed a significant increase in RSN identifiability (*p* < 0.0001, see Fig. 1). Similar results were obtained using different thresholds to generate the RSNs mask and using ICA-AROMA and FIX aggressive option to remove components (see Supplementary Material Fig. 2).

### 3.4 RSN reproducibility

ICA-AROMA and patient-trained FIX yielded significantly higher RSN reproducibility when compared to pre-processed uncorrected (*p* < 0.0001), though no significant difference was found between ICA approaches (Fig. 1).

### 3.5 Loss in temporal degrees of freedom

The loss in tDoF was significantly different across ICA approaches (*p* < 0.001, Fig. 1). The loss in tDoF was greatest when using patient-trained FIX (mean 35%, SD 7%) and lowest when using ICA-AROMA (mean 25%, SD 3%). The mean total variance of the components labelled as noise using FIX was 75% (SD 11%) and 71% using ICA-AROMA (SD 9%).

### 3.6 Effect of correction on BOLD variance

**Fig. 2:**
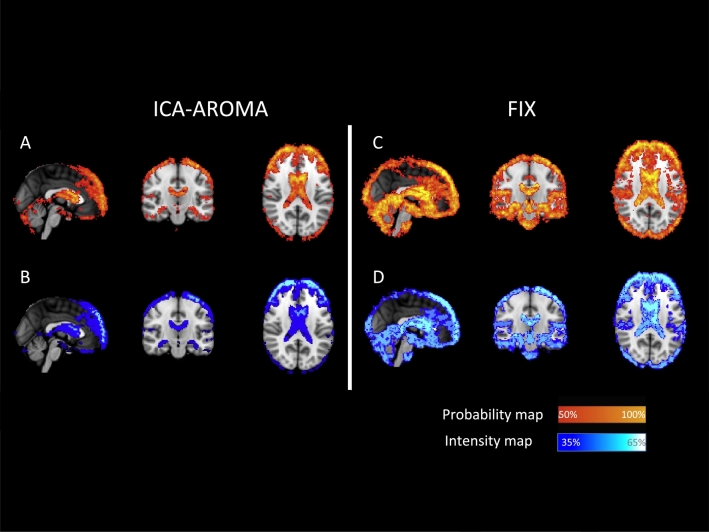
Spatial pattern of change in BOLD signal standard deviation after using patient-trained FIX and ICA-AROMA. (A, C) Probability maps, representing areas where the BOLD variance was affected more frequently in patients. (B, D) Intensity maps, representing areas where voxels %ΔSTD was significantly more reduced in patients.

**Fig. 3:**
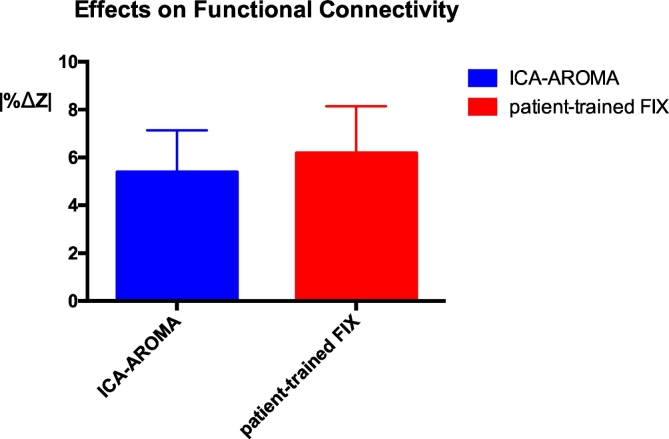
Effects of ICA-AROMA and patient-trained FIX on resting state connectivity estimation. After performing a template-based dual regression analysis on the original data and on the data cleaned using ICA-AROMA and patient-trained FIX, differences in estimation of functional connectivity were evaluated using |%ΔZ |. *** *p* < 0.001.

When using ICA-AROMA, individual average maps of %ΔSTD showed more frequent and more pronounced changes around the brain edges and in the periventricular areas with an average decrease of 35–40% in the BOLD signal variance (Fig. 2A and 3B).

When using patient-trained FIX, the changes were more widespread and were particularly pronounced in the inter-sulci regions spreading to the central, pre-cuneal, insular, and paracingulate areas (Fig. 2C). On average, pre-processing the data with FIX led to a consistent and broad decrease of 40–45% in the BOLD signal variance. A further decrease (up to 55%) of the variance was confined to the periventricular-perivascular areas (Fig. 2D).

### 3.7 Effect of correction on functional connectivity

Denoising data using patient-trained FIX was associated with significantly higher changes in the intra-network connectivity compared to ICA-AROMA (*p* < 0.001, Fig. 3). The mean absolute functional connectivity changes across subjects and across networks was 5.3% (SD 2.1%) using ICA-AROMA and 6.2% (SD 2.6%) when using patient-trained FIX. |%ΔZ | did not differ significantly across the NOIs when using either ICA-AROMA or FIX.

**Fig. 4:**
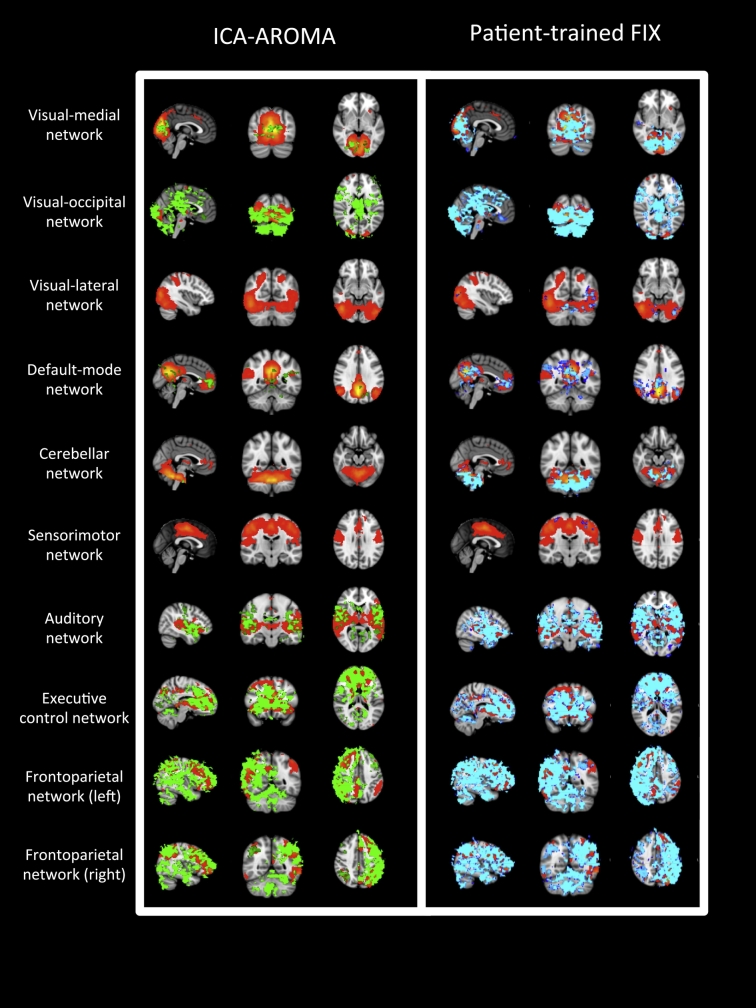
Group changes in functional connectivity in the 10 “well defined” networks. For each of the 10 “well defined” networks (red-yellow), significant group level changes (*p* < 0.05 TFCE) in functional connectivity are shown after the use of ICA-AROMA (green) and after the use of patient-trained FIX (blue). From top to bottom: visual-medial, visual-occipital, visual-lateral networks, the default mode network, the cerebellar network, the sensorimotor network, the auditory network, the executive-control and the left and right fronto parietal networks. (For interpretation of the references to color in this figure legend, the reader is referred to the online version of this chapter.)

The spatial maps representing significant group-level changes in functional connectivity (*p* < 0.05 TFCE) after applying ICA-AROMA and patient-trained FIX in the 10 “well defined” networks are shown in Fig. 4. Changes affected a higher number of voxels when using patient-trained FIX compared to ICA-AROMA, particularly in the visual-medial, default mode, auditory, and cerebellar networks.

### 3.8 Grayplots

Twenty grayplots (selected at random from the 60 possible) are presented in the Supplementary Material (see Supplementary Material Fig. 3: 1–20). Green lines and blue lines indicate voxels in the white and gray matter, respectively. Motion artefacts (dark vertical bands affecting both gray and white matter) appear to be attenuated, but not completely removed by the use of either ICA-AROMA or patient-trained FIX.

## 4 Discussion

ICA based denoising approaches were successfully applied to rs-fMRI data obtained from acute stroke patients resulting in improved RSN reproducibility, localized reduction in BOLD variance consistent with noise removal and significant changes in functional connectivity. Compared to ICA-AROMA, patient-trained FIX resulted in higher RSN identifiability and wider changes both in the BOLD variance and in functional connectivity.

The use of all ICA strategies led to a significant increase in RSNs reproducibility reflecting the reduction of the group level variability due to noise. Following the use of ICA-AROMA, changes in the BOLD signal variance were both more frequent and more prominent in proximity of the brain edges, consistent with the intended removal of motion-related artefact. The changes in BOLD signal variance after the use of patient-trained FIX were more extensive, also involving regions in proximity of large vessels (particularly around the sagittal sinus) suggesting the ability to capture and remove physiological noise (vascular and CSF pulsation artefacts).

The removal of noise may come at a cost. Decreased tDoF might undermine the reliability of subject-level functional connectivity estimates and introduce a bias in group-level analyses due to between-subject tDoF-variability (Birn et al., 2013, Yan et al., 2013a, Yan et al., 2013b, Pruim et al., 2015b). This study demonstrates that both ICA-AROMA and patient-trained FIX result in lower or similar loss of tDoF when compared with other motion correction strategies such as spike regression, scrubbing, and 24 realignment parameters (Pruim et al., 2015b). This suggests that any bias introduced through noise removal is limited and in-keeping with that expected when using other approaches.

When it came to the assessment of the preservation of the signal of interest, this study demonstrated findings consistent with previous results following the use of ICA-AROMA (Pruim et al., 2015b), confirming its robustness and generalizability. Removal of noise had an impact on the estimation of intra-network connectivity in all patients associated with widespread changes in mean estimated functional connectivity.

The use of generic-trained FIX in this acute stroke population proved to be a non-viable choice due to significant issues with misclassified and discarded signal components. The FIX generic training weight dataset was developed on an identical scanner and using identical acquisition protocols to those used in this study. These issues with FIX accuracy, therefore, suggest the presence of disease specific effects altering both noise and signal components and that these effects are at least as important as those introduced by either scanner or acquisition protocol.

This study demonstrates the necessity of the appropriate training of FIX. Appropriate training of FIX in this specific cohort needs to take account of the dynamics of both noise and signal components over the first month following stroke. This will include the transition from the effects of injury in the first scans to the effects of recovery and plasticity seen in the later scans. The patient-training dataset was developed using data from a broad range of patients from all time points across the first month following acute stroke. Its use resulted in an increase in FIX accuracy, not least through the increase in RSN identifiability reflecting higher signal to noise levels, even in a population with such wide and heterogeneous sources of variance as acute stroke patients.

The approach of appropriate training needs validation in different disease processes. We make available our patient-training dataset (http://www.fmrib.ox.ac.uk/analysis/FIX-training/) for testing in separate populations of acute stroke patients. If healthy controls are to be included in an analysis, a different training dataset will likely be required (Griffanti et al., 2016). It is worth noting that the components identified as noise using FIX in this study (where the focus was acquiring data to use resting-state BOLD fluctuations as an indicator of neural activity) may present useful information in different contexts for the interrogation of cerebrovascular pathology (Webb and Rothwell, 2016).

Both a focused (ICA-AROMA) and a complex (patient-trained FIX) classifier approach result in significant noise removal from rs-fMRI data acquired from a population of acute stroke patients. The success of ICA-AROMA demonstrates that by concentrating on a restricted number of components related to a very common cause of noise, namely motion, the full automation of an ICA-based approach can deliver meaningful data for analysis even in population with such heterogeneous sources of noise. However, the time invested to train FIX with appropriate patient data encompassing the multiple sources of disease-specific noise (in addition to those that are scanner- and acquisition-related) proved valuable, particularly in improving the signal-to-noise ratio. Depending on the application, both approaches clearly have a role in future stroke studies: ICA-AROMA might be preferred in the setting of noise removal from rs-fMRI data in a multi-centre study where individual site patient numbers might be low or the expertise to develop the training dataset might be limited (both impeding the development of a site-specific training dataset), and the scanners and acquisition protocols might be varied across sites; whereas, patient-trained FIX might be have a role in individual proof of concept studies where the resulting improvement in signal-to-noise ratio means that committing resources to training FIX appropriately are important.

## Appendix A Supplementary data

Supplementary figures 1-3 Image 1
