# Supplementary material for: Impact of automated ICA-based denoising of fMRI data in acute stroke patients
Source: Neuroimage Clin. 2017 Jun 30;16:23–31. doi: 10.1016/j.nicl.2017.06.033 (PMC5508492; doi:10.1016/j.nicl.2017.06.033)
Supplement: Supplementary file 1 — Supplementary figures 1-3 [file mmc1.pdf]

Supplementary Material

- Supplementary Figure 1. Z-scores variance across voxels.
- Supplementary Figure 2. Resting State Network Identifiability.
- Supplementary Figure 3 (1-20). Grayplots.

Supplementary Figure 1**. Z-score distribution across voxels**. Representative histograms of the distribution of the Z-scores across voxels (single scan level) for each of the "10 well defined" components, before (red) and after the use of ICA-AROMA (blue) and patient-trained FIX (green).

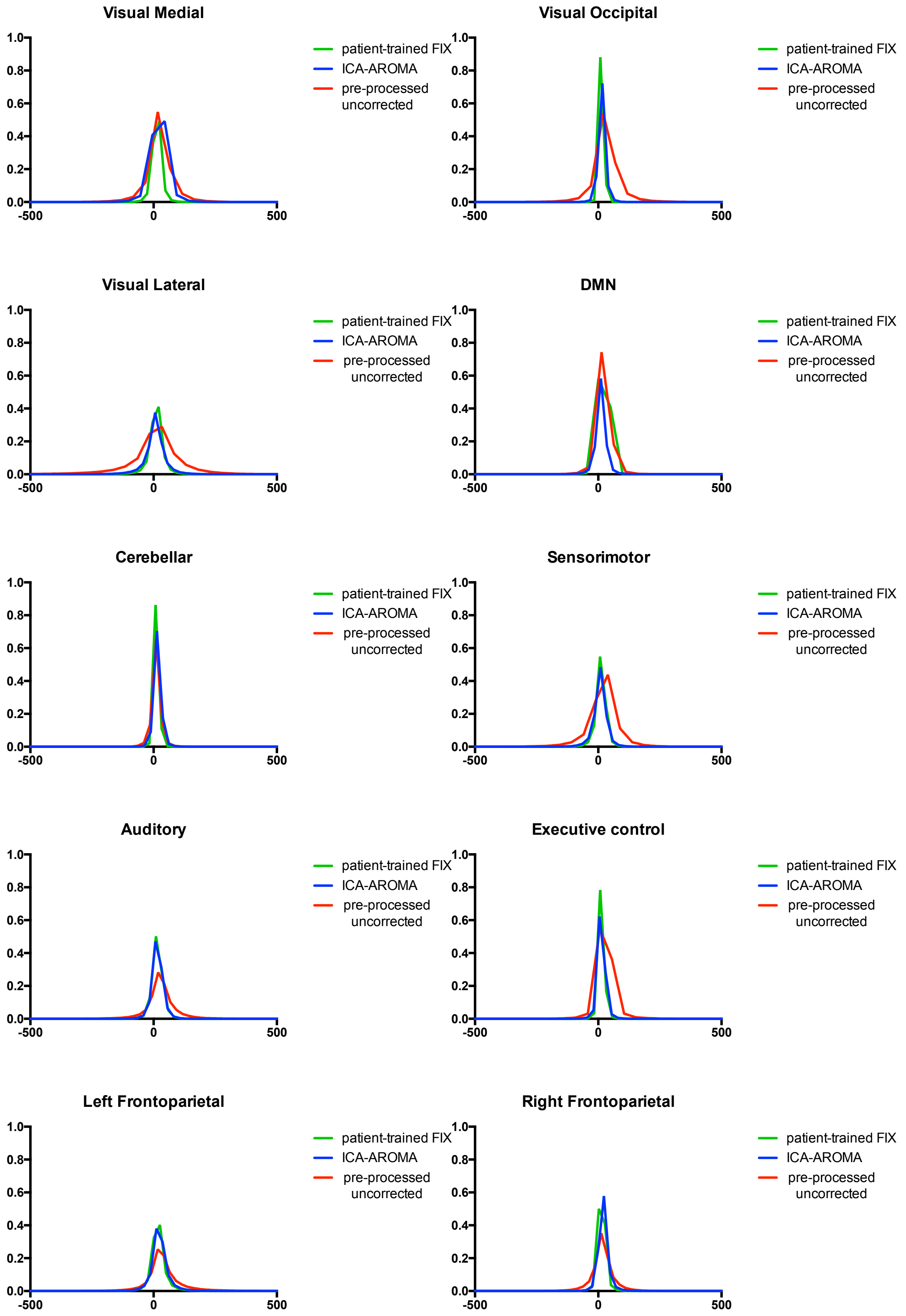

Supplementary Figure 2**. RSN Identifiability**. RSN identifiability analysis using a more lenient threshold (|Z|>1.5) and a more conservative threshold (|Z|>3.1) for the creation of the RSN masks (left and middle) and after regressing the artefactual components using the aggressive option (right).

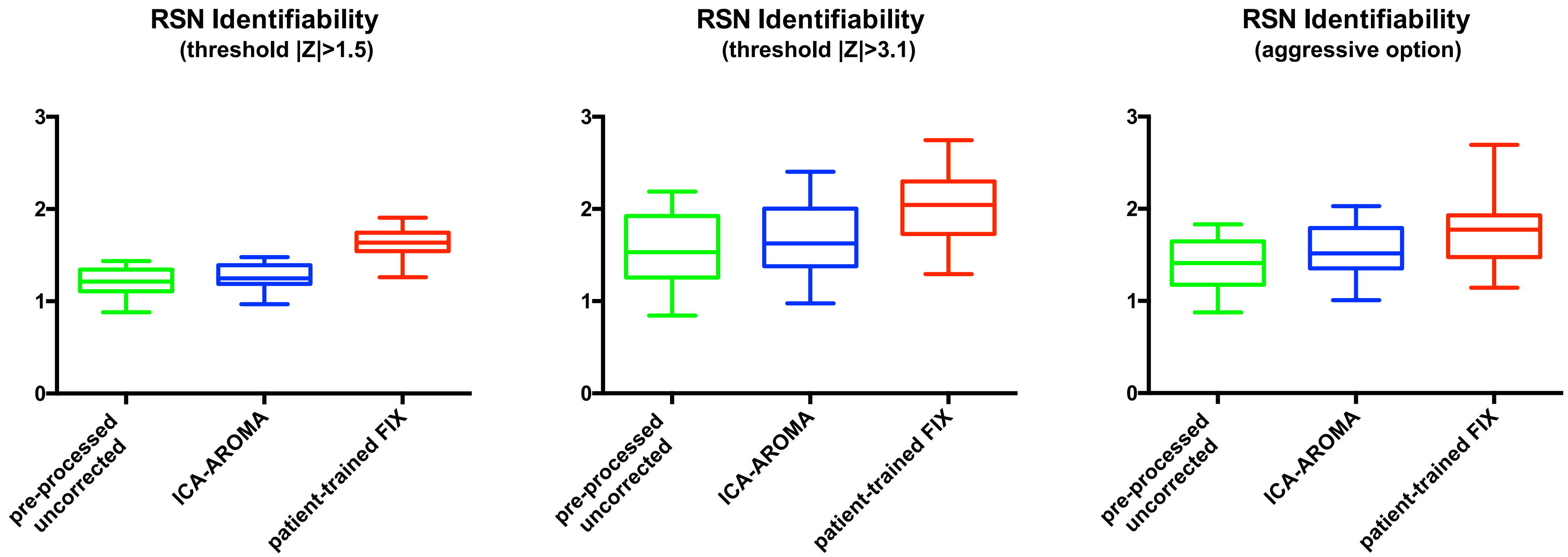

Tukey's multiple comparisons test	Mean Diff.	95% CI of diff.	Significant?	Summary
pre-processed uncorrected vs. ICA-AROMA	-0.06289	-0.1790 to 0.05324	No	ns
pre-processed uncorrected vs. patient-trained FIX	-0.4250	-0.5427 to -0.3074	Yes	****
ICA-AROMA vs. patient-trained FIX	-0.3621	-0.4798 to -0.2445	Yes	****

Tukey's multiple comparisons test	Mean Diff.	95% CI of diff.	Significant?	Summary
pre-processed uncorrected vs. ICA-AROMA	-0.1049	-0.3934 to 0.1836	No	ns
pre-processed uncorrected vs. patient-trained FIX	-0.4678	-0.7601 to -0.1755	Yes	***
ICA-AROMA vs. patient-trained FIX	-0.3629	-0.6552 to -0.07063	Yes	*

Tukey's multiple comparisons test	Mean Diff.	95% CI of diff.	Significant?	Summary
pre-processed uncorrected vs. ICA-AROMA	-0.1282	-0.3577 to 0.1014	No	ns
pre-processed uncorrected vs. patient-trained FIX	-0.3474	-0.5770 to -0.1179	Yes	**
ICA-AROMA vs. patient-trained FIX	-0.2192	-0.4488 to 0.01032	No	ns

Supplementary Figure 3 (1-20)**. Grayplots**. Grayplots of 20 scans (randomly selected) displaying at a single-acquisition level the effects of using ICA-AROMA and patient-trained FIX. Green and blue lines indicate voxels in the white and gray matter, respectively.

1

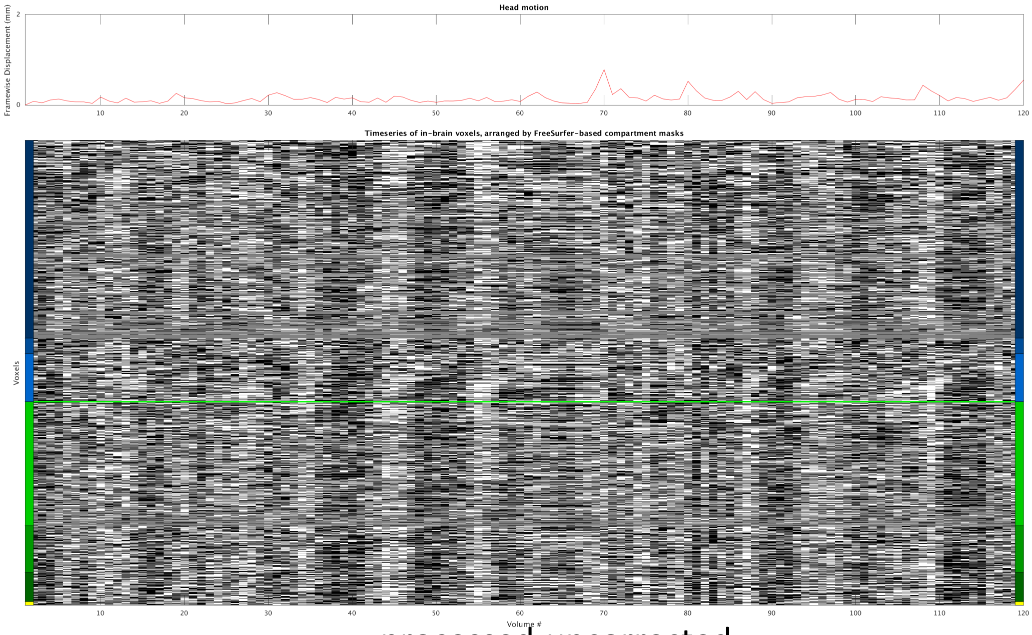

processed-uncorrected

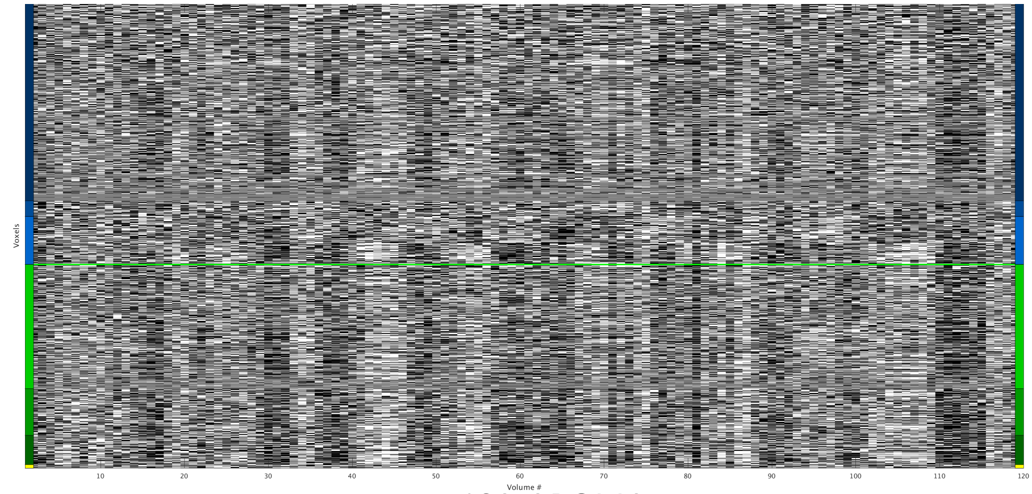

ICA-AROMA

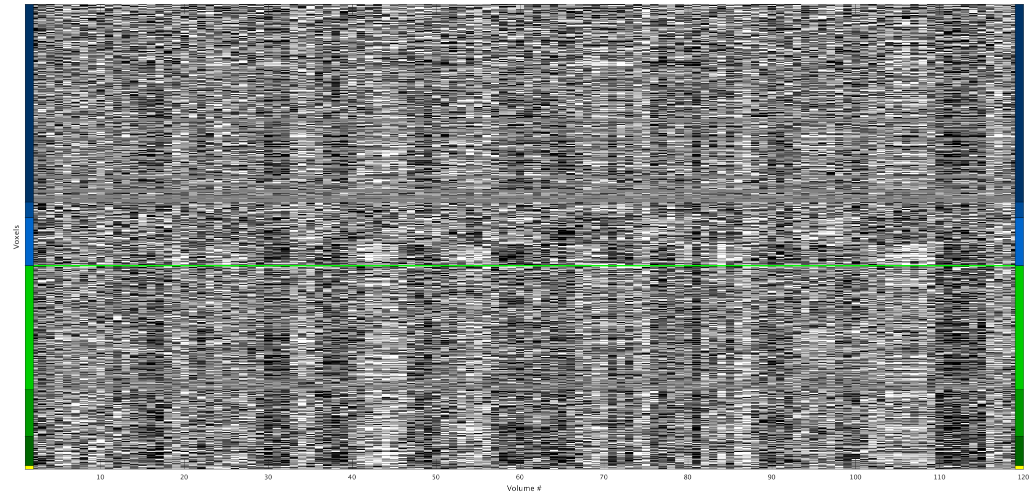

patient trained FIX

2

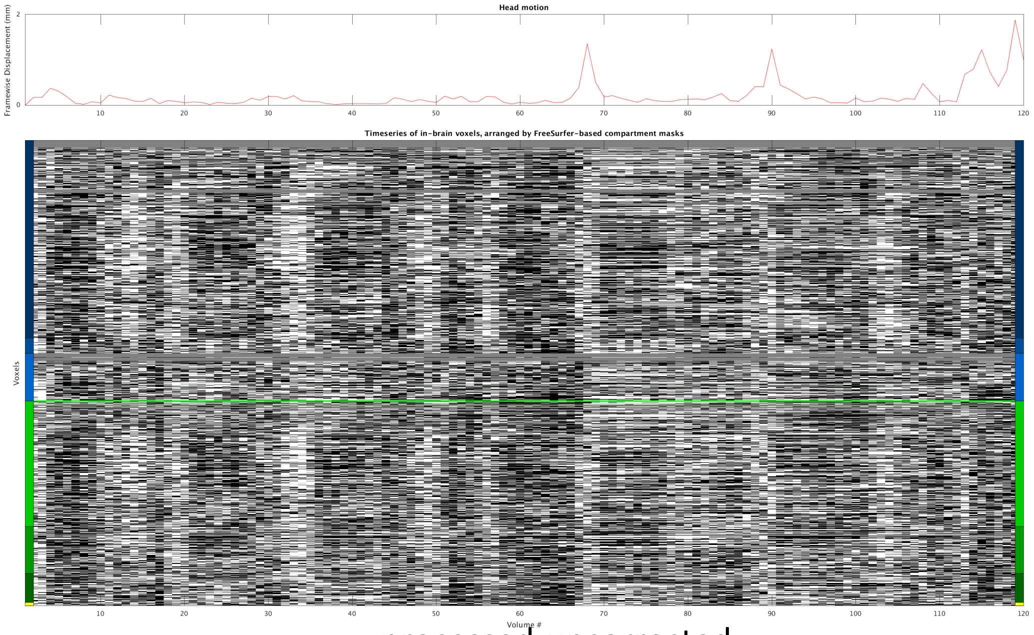

processed-uncorrected

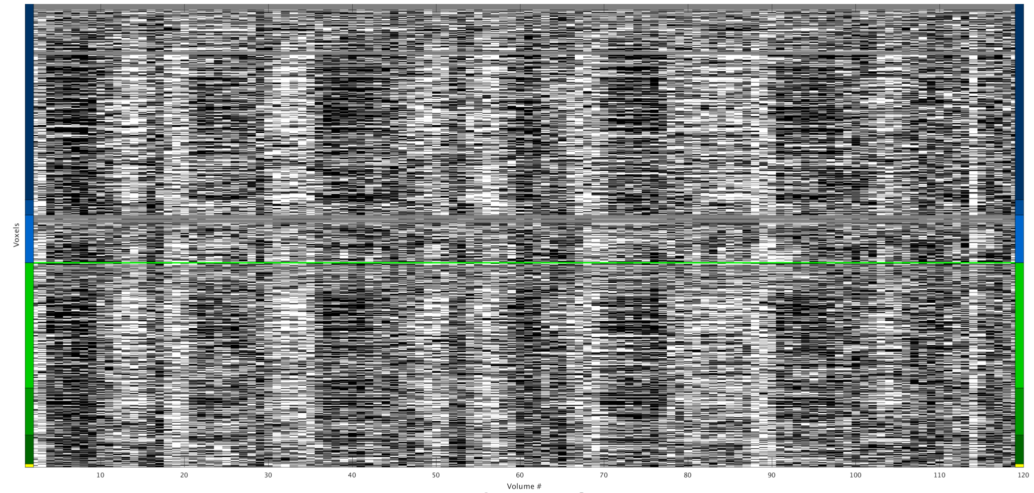

ICA-AROMA

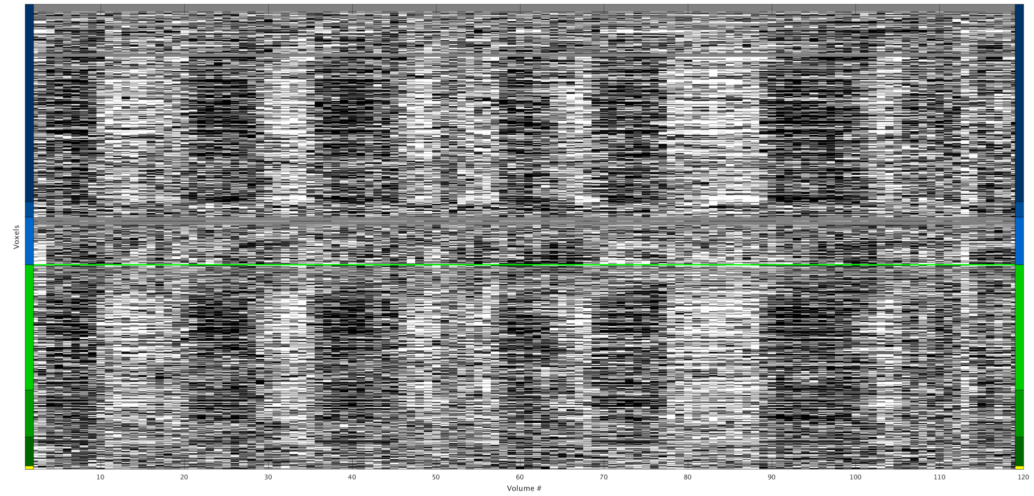

patient trained FIX

3

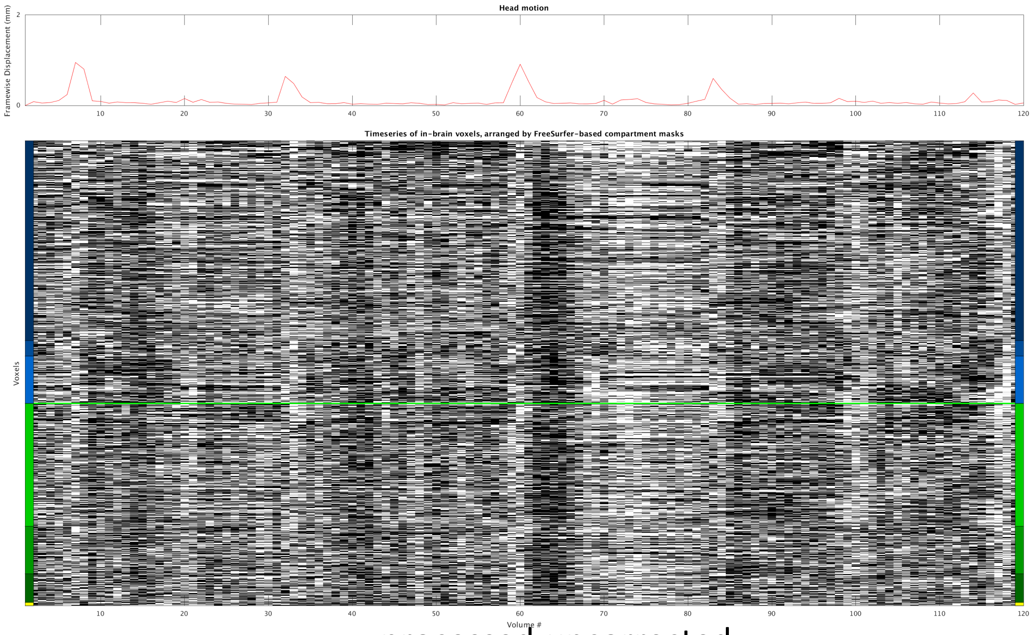

processed-uncorrected

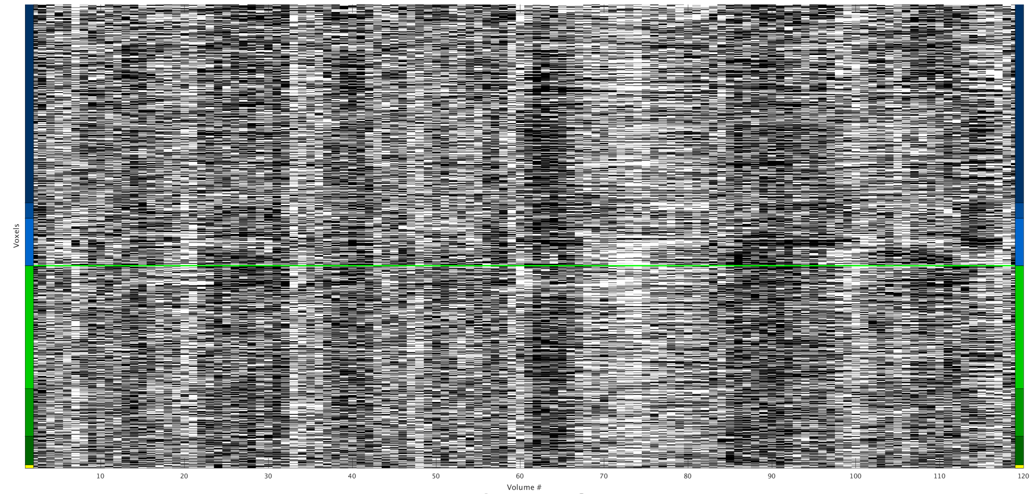

ICA-AROMA

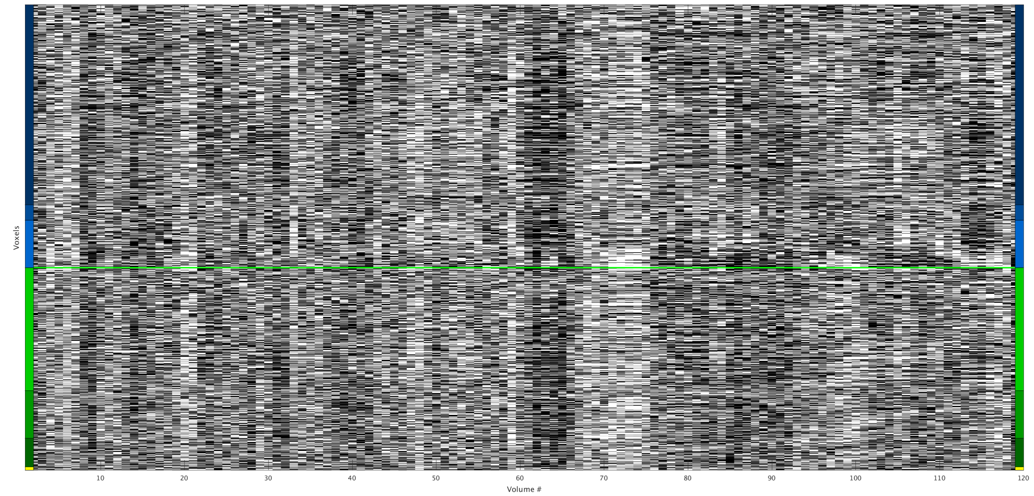

patient trained FIX

4

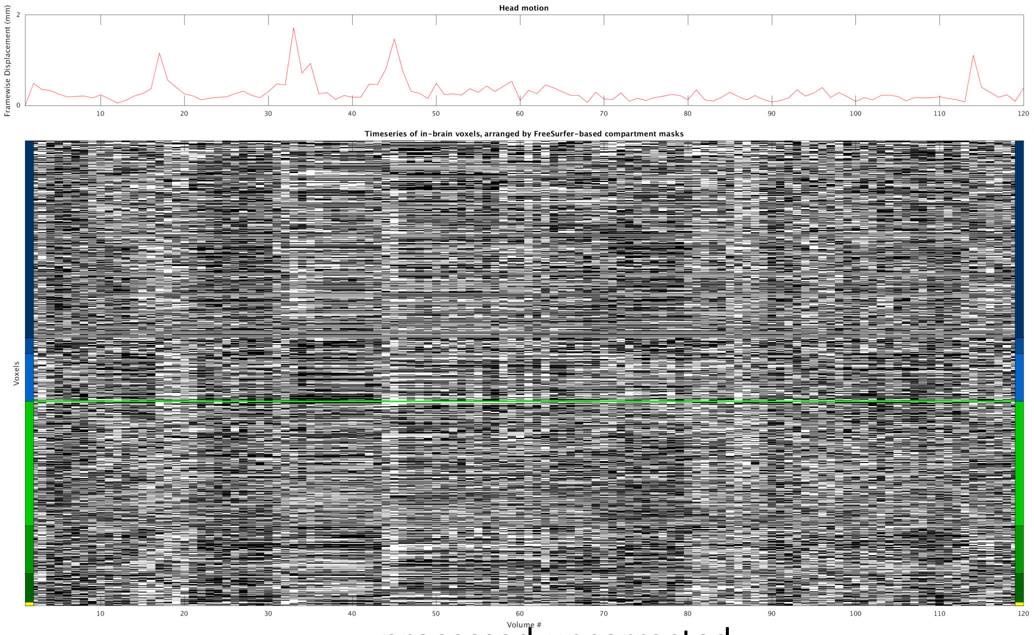

processed-uncorrected

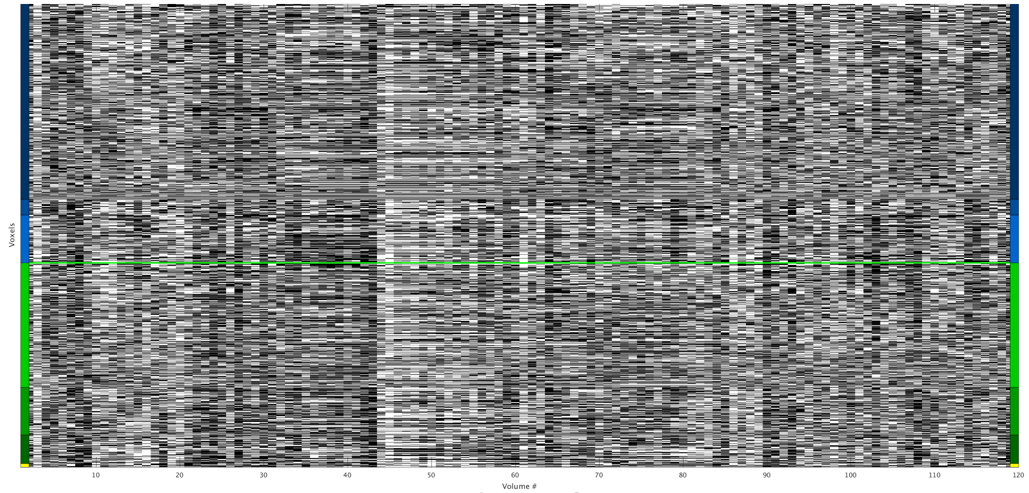

ICA-AROMA

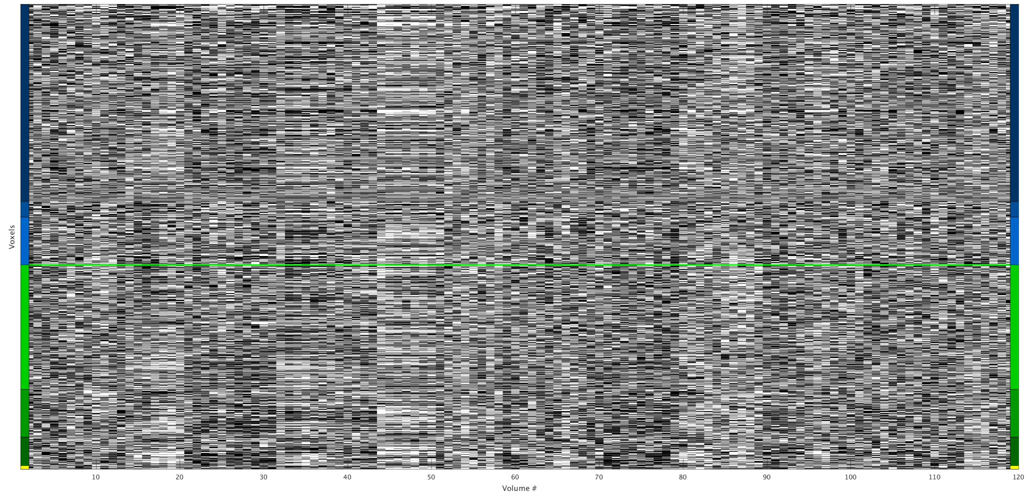

patient trained FIX

5

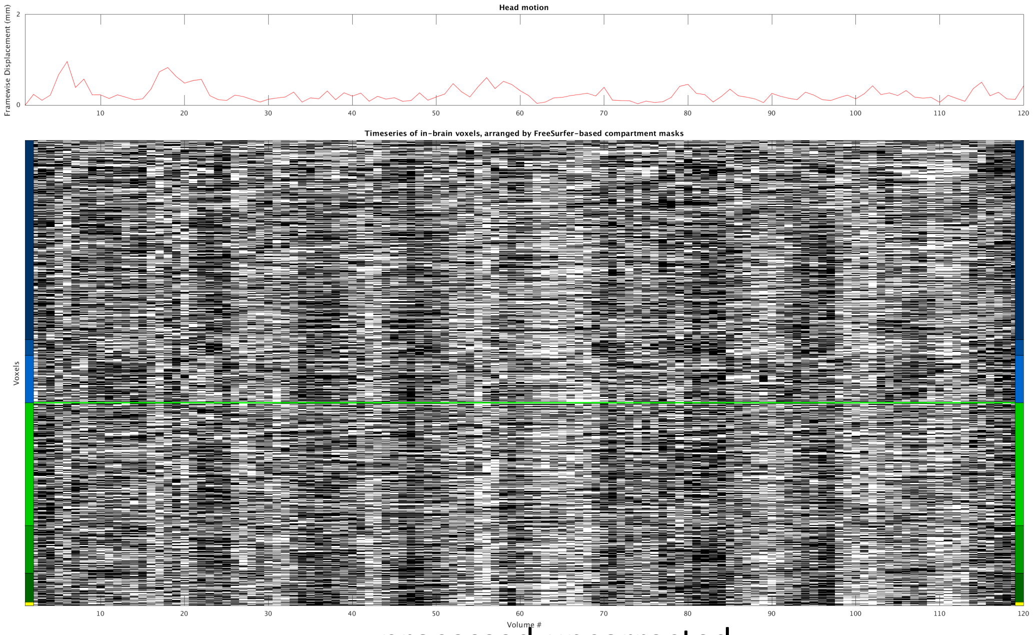

processed-uncorrected

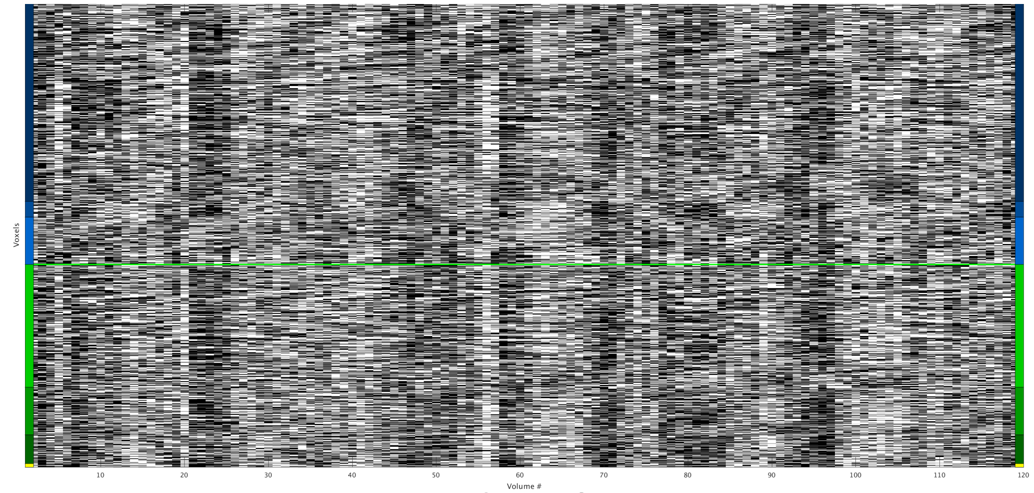

ICA-AROMA

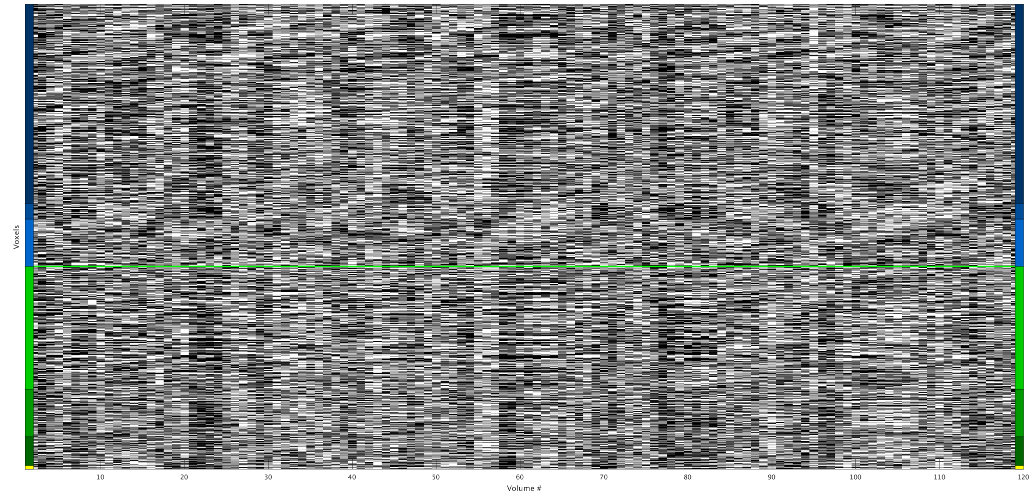

patient trained FIX

6

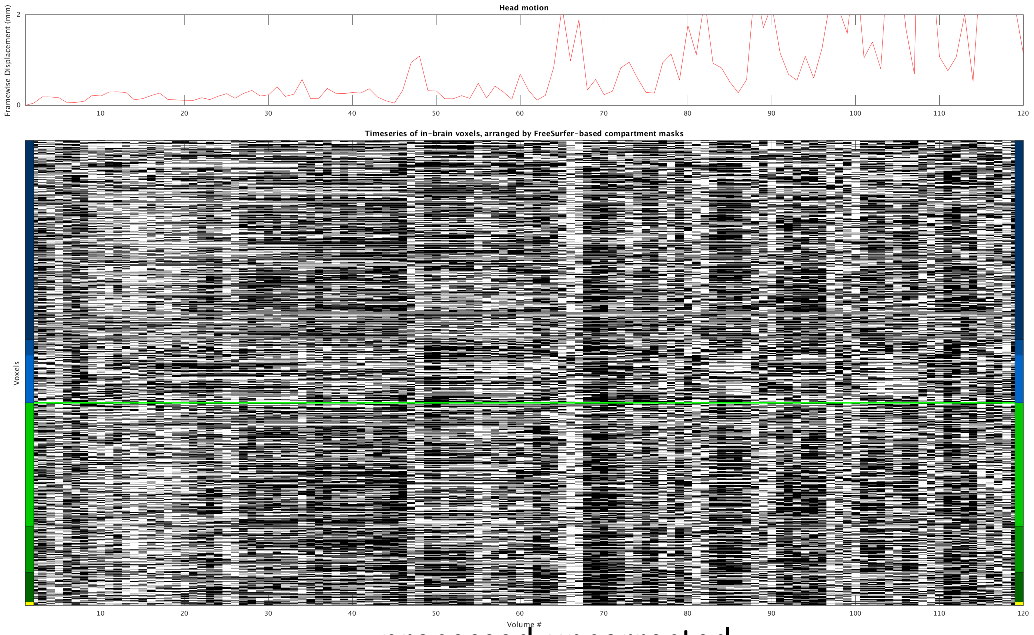

processed-uncorrected

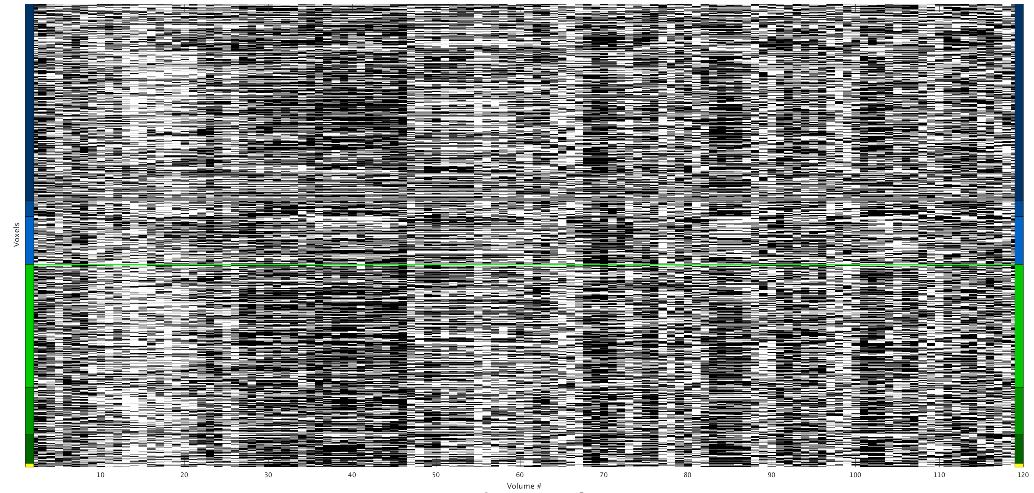

ICA-AROMA

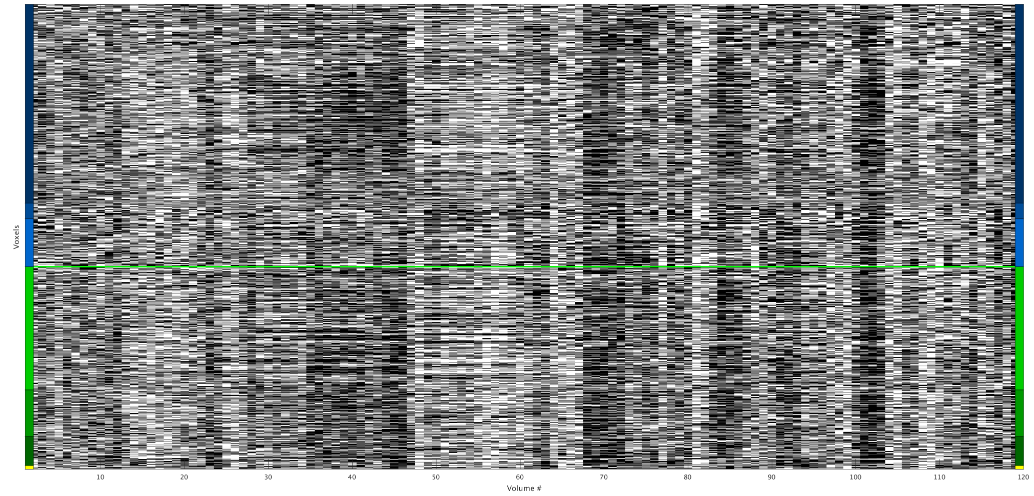

patient trained FIX

7

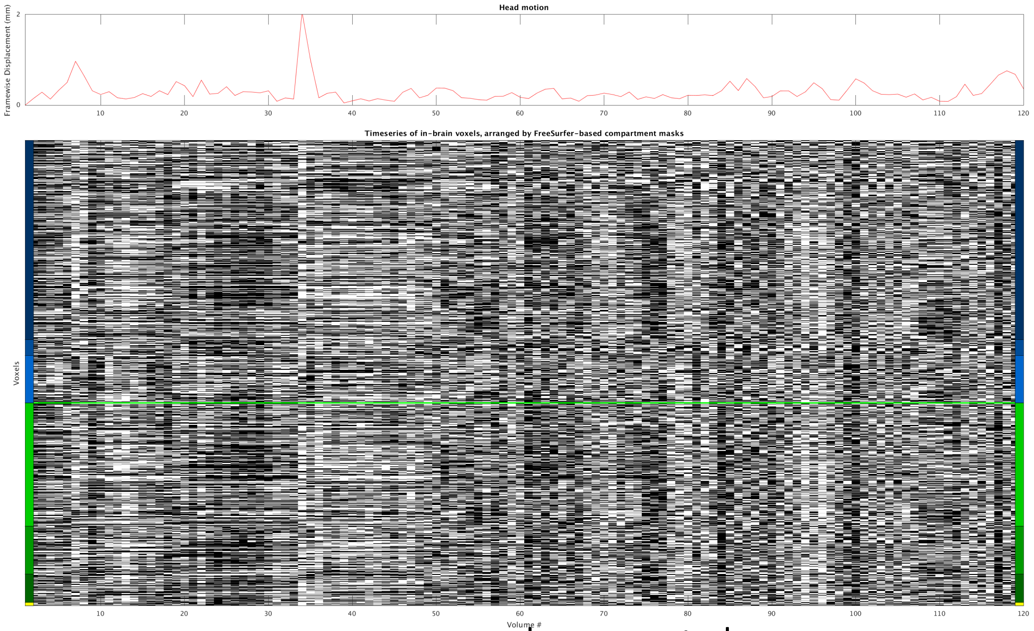

processed-uncorrected

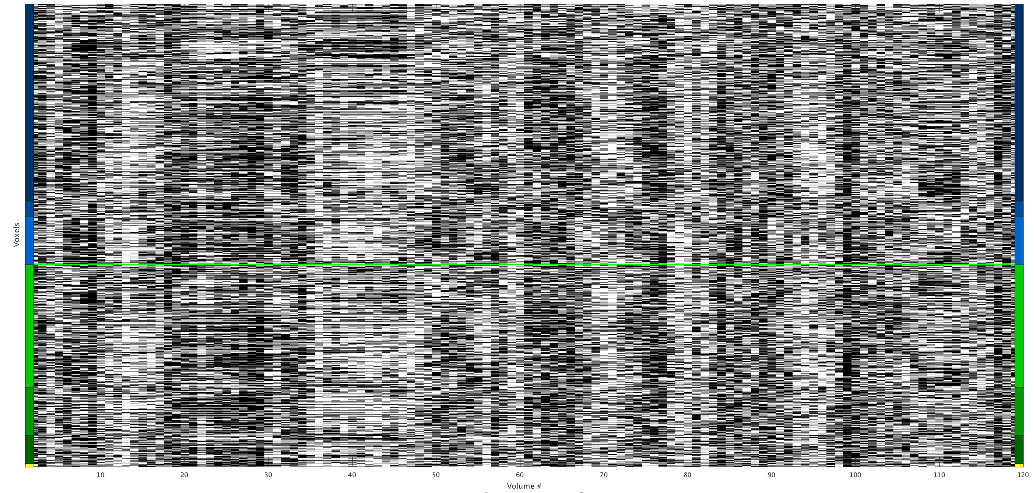

ICA-AROMA

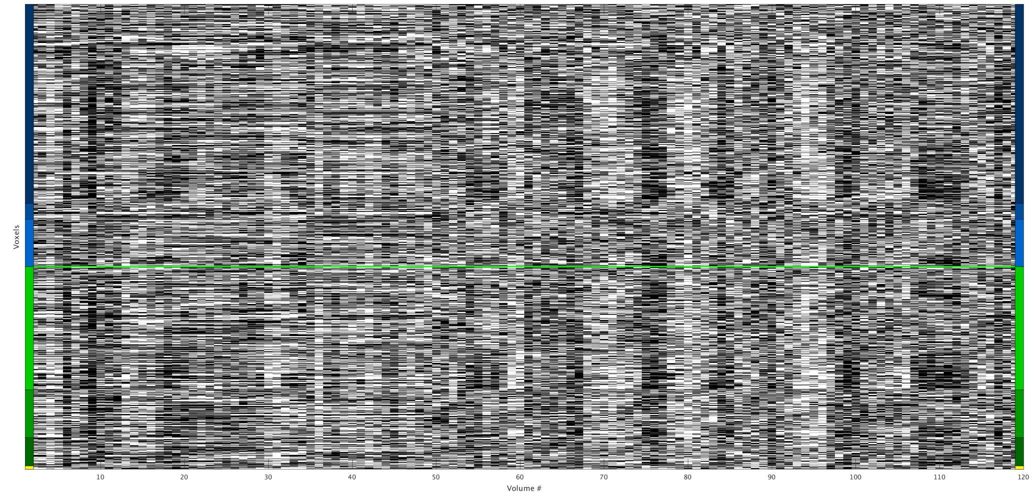

patient trained FIX

8

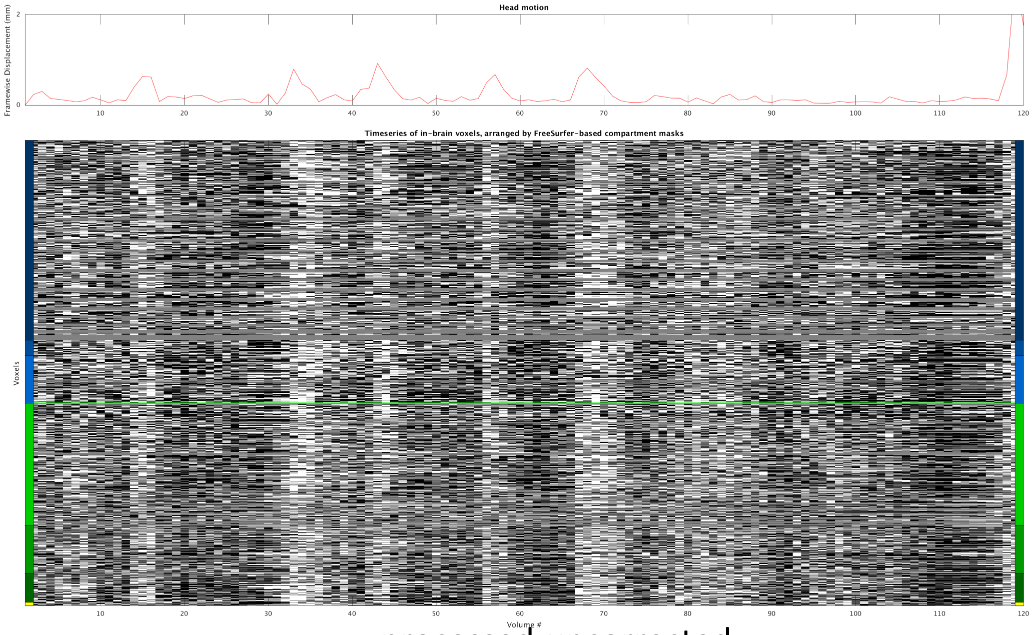

processed-uncorrected

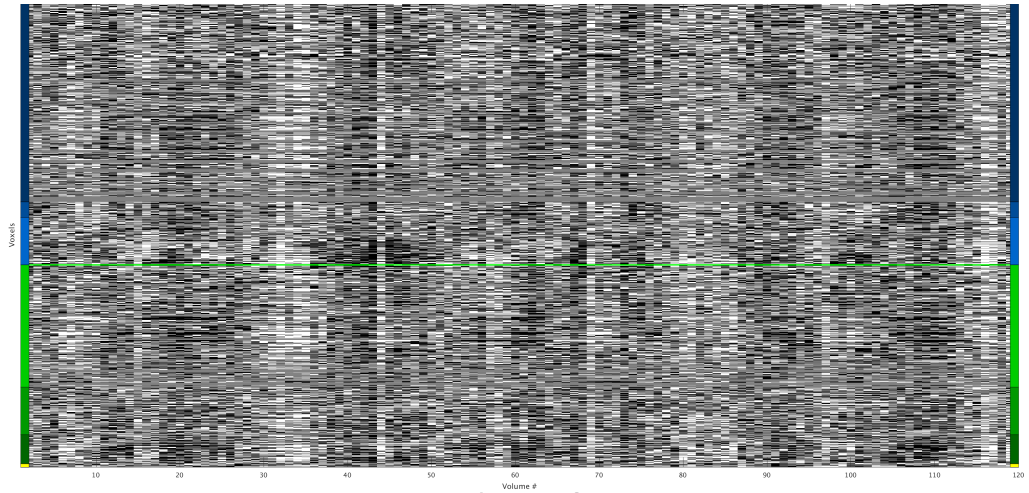

ICA-AROMA

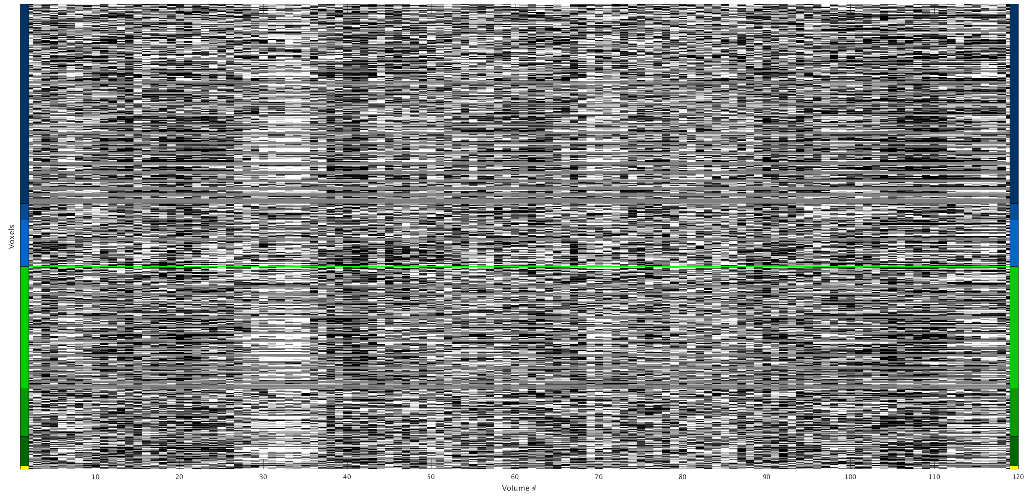

patient trained FIX

9

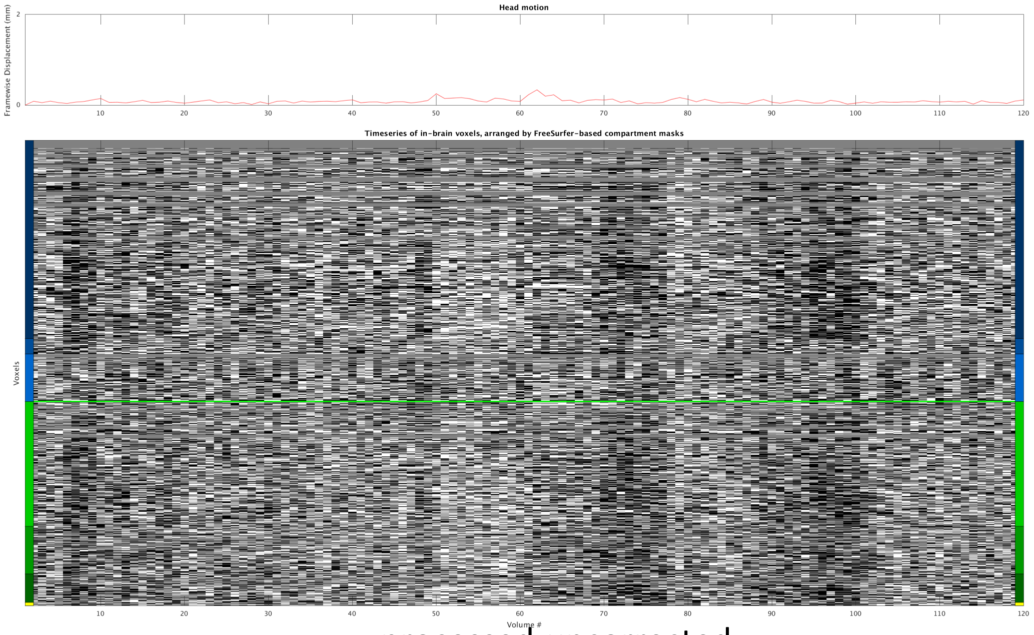

processed-uncorrected

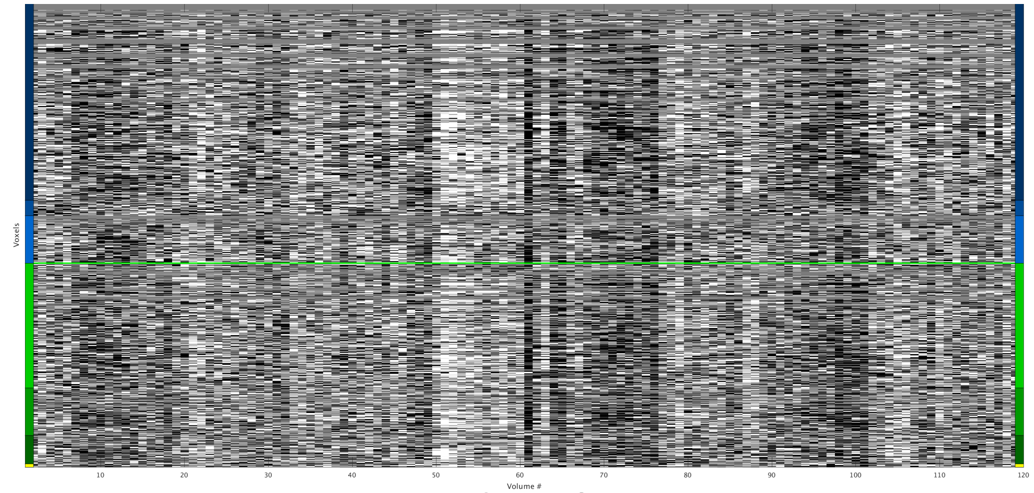

ICA-AROMA

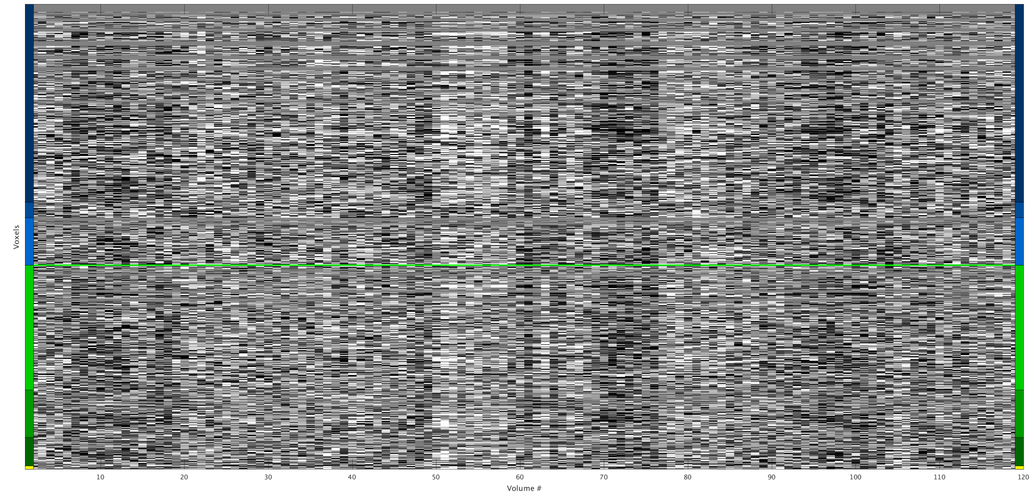

patient trained FIX

10

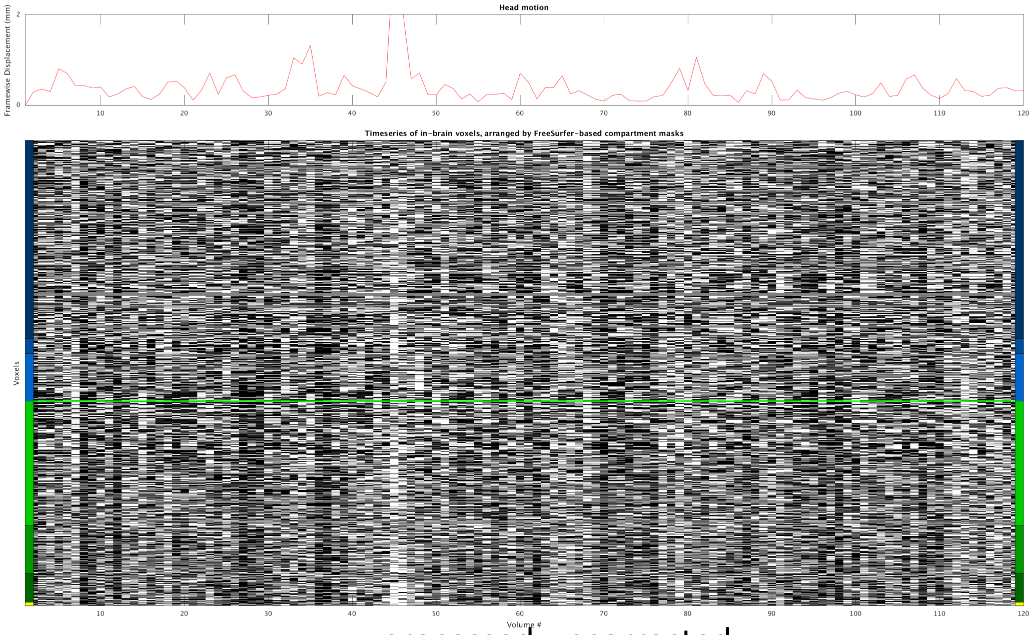

processed-uncorrected

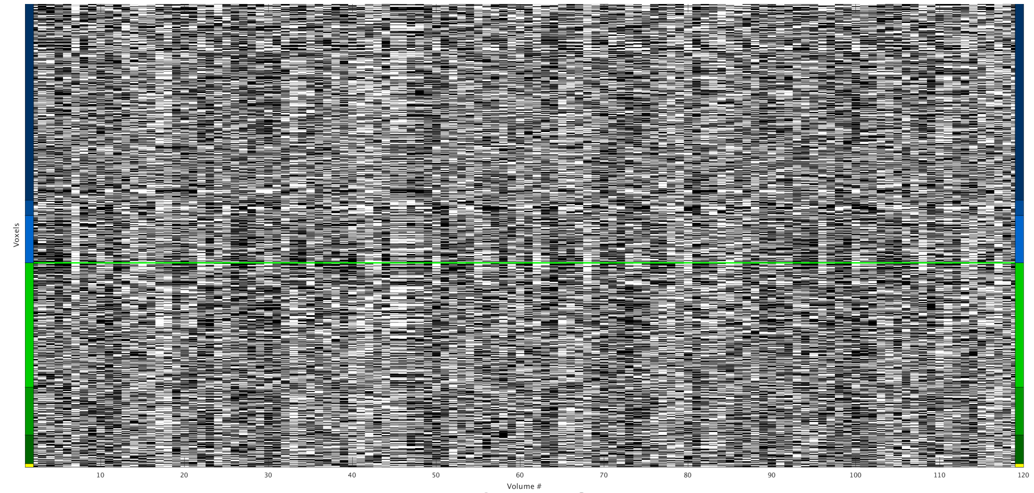

ICA-AROMA

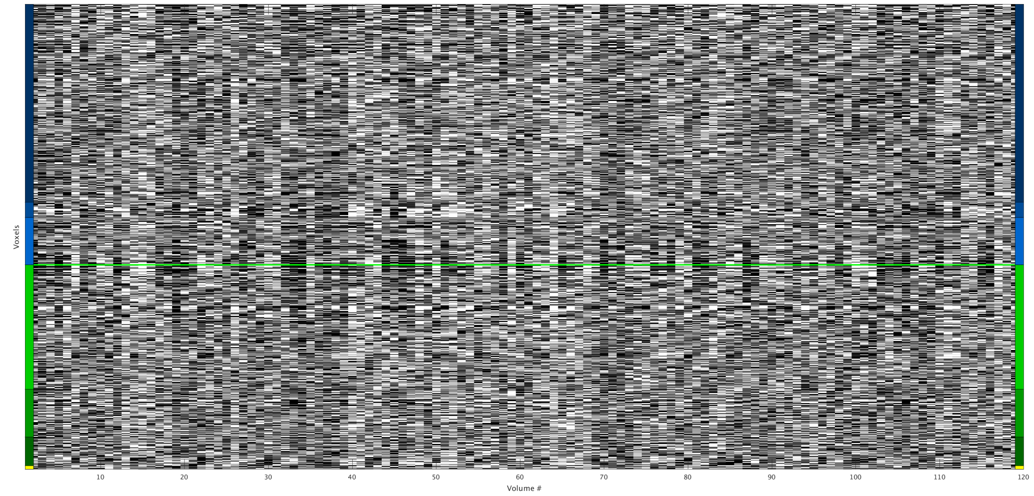

patient trained FIX

11

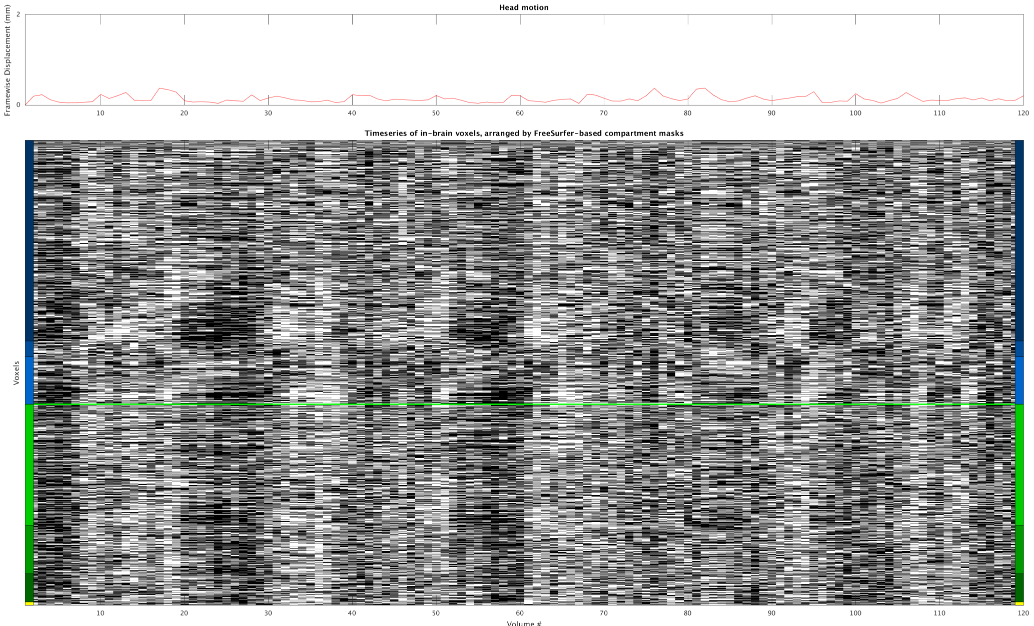

processed-uncorrected

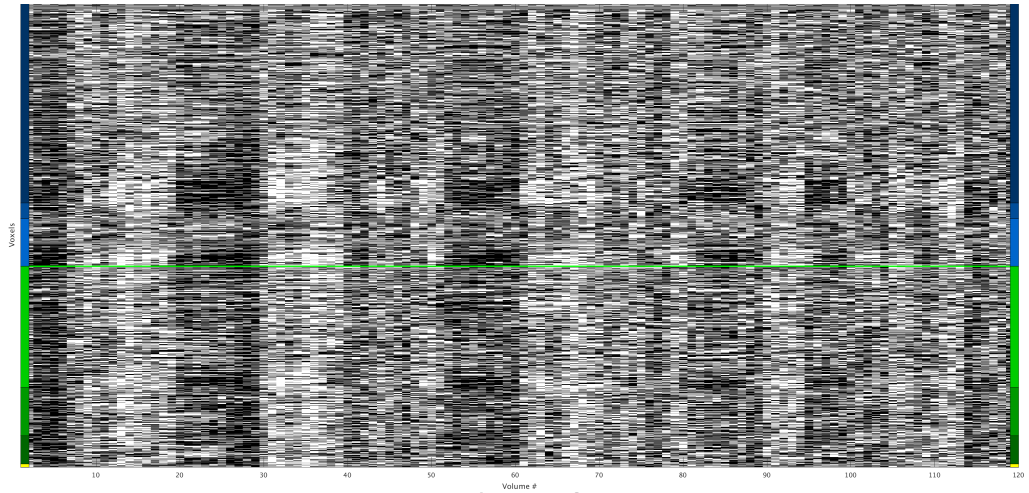

ICA-AROMA

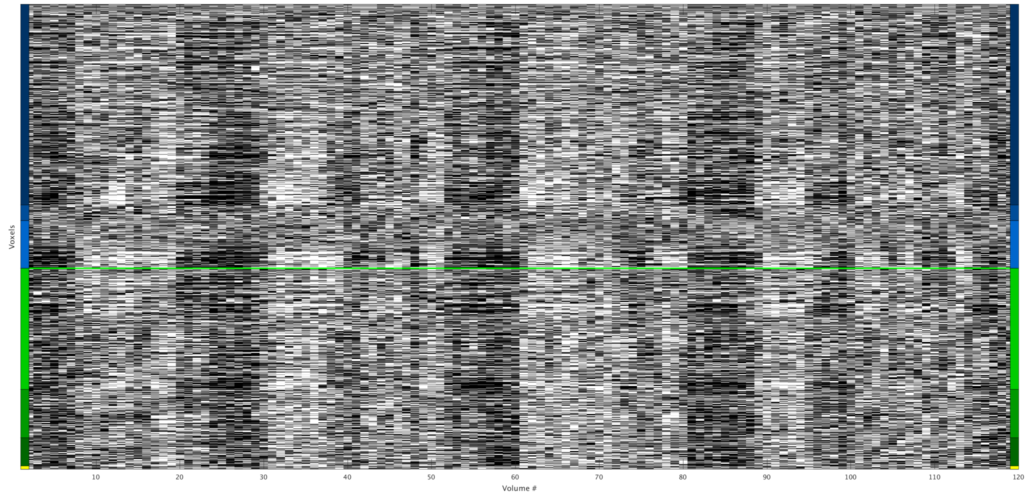

patient trained FIX

12

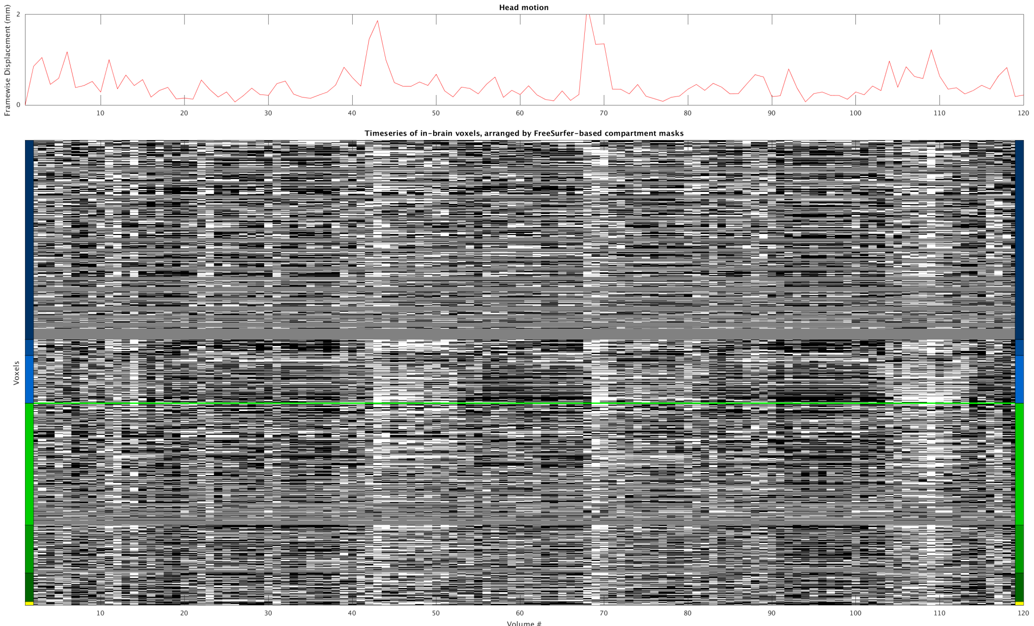

processed-uncorrected

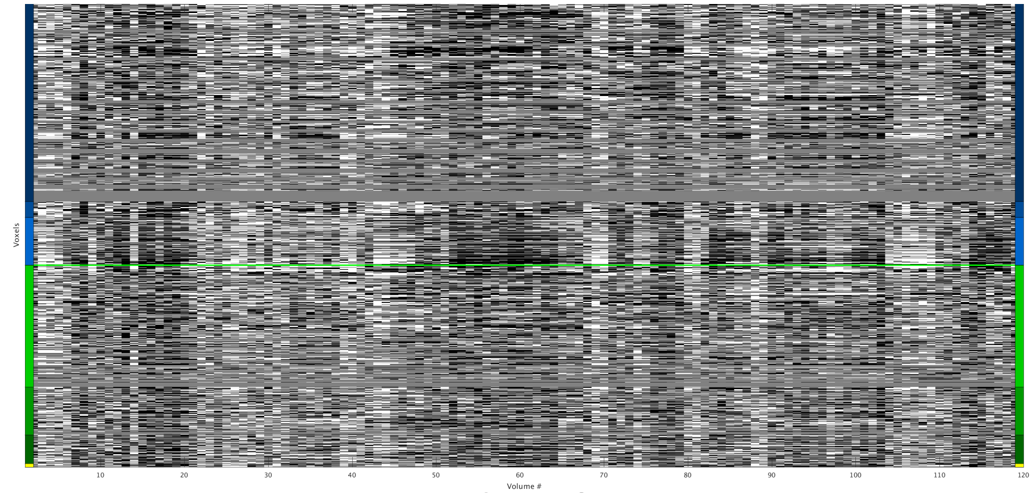

ICA-AROMA

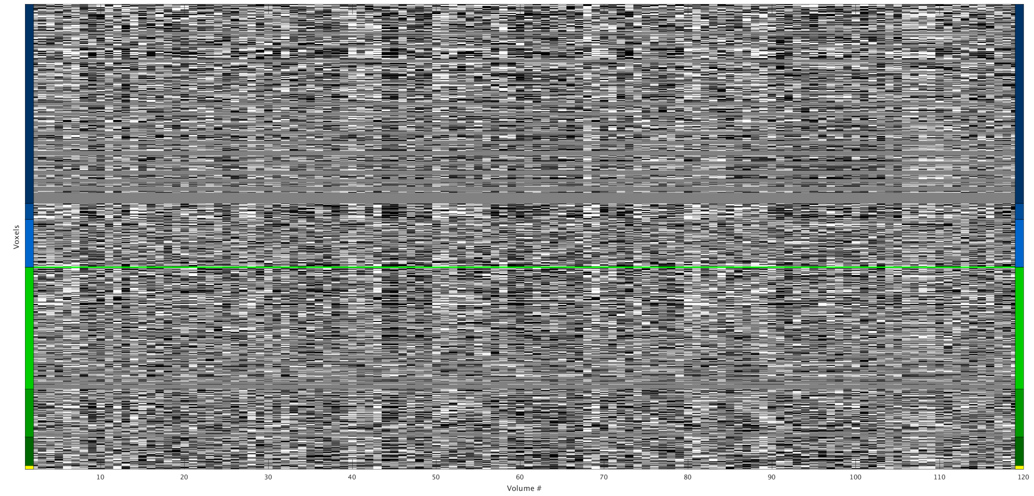

patient trained FIX

13

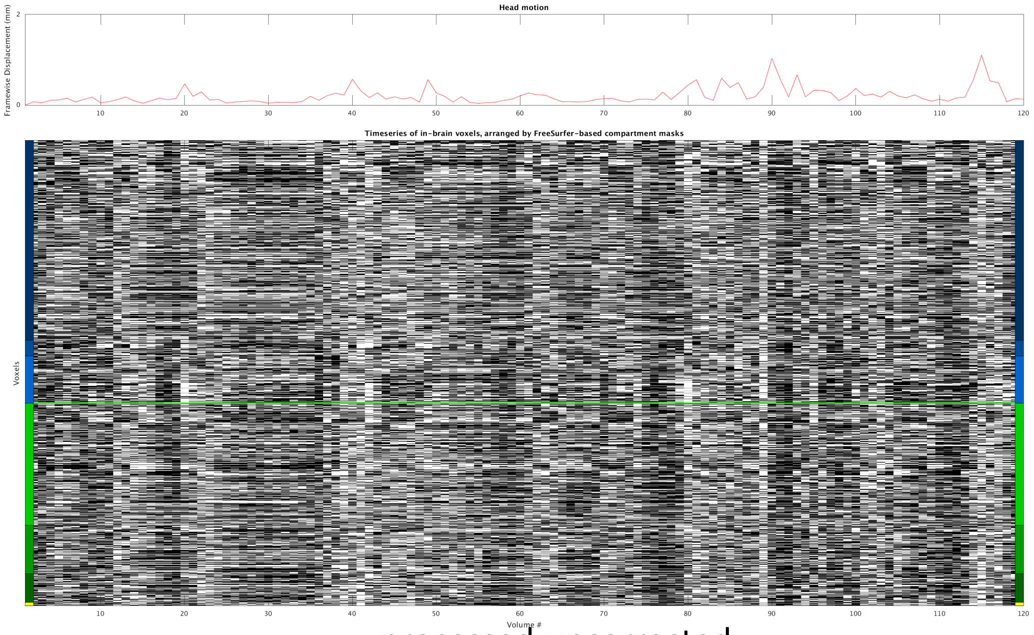

processed-uncorrected

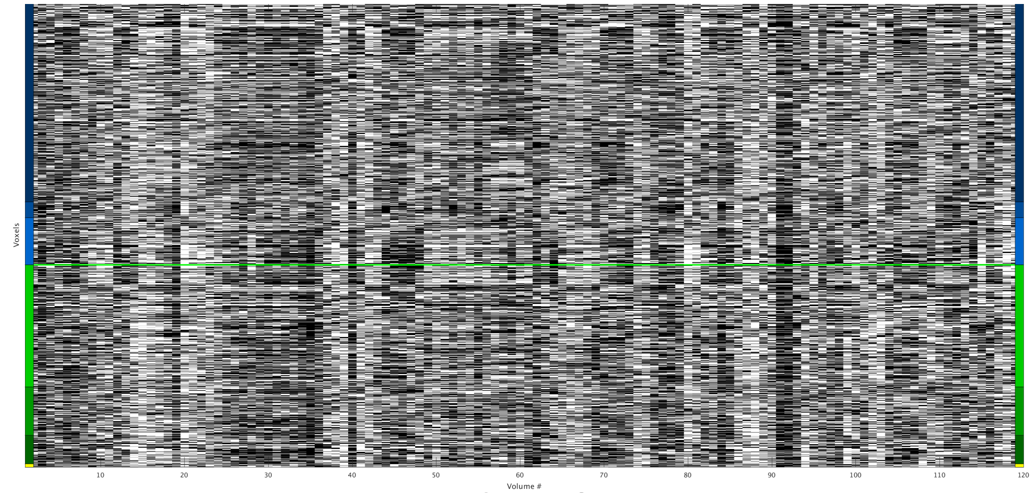

ICA-AROMA

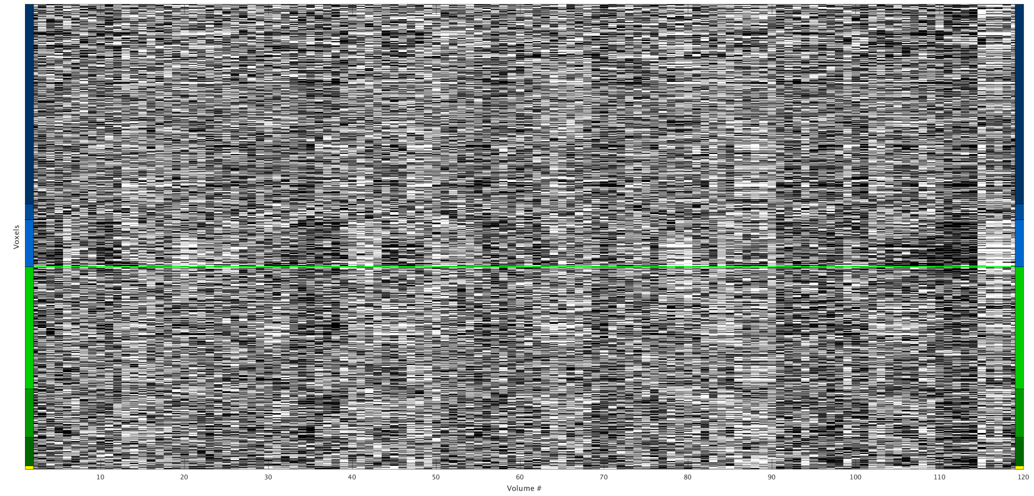

patient trained FIX

14

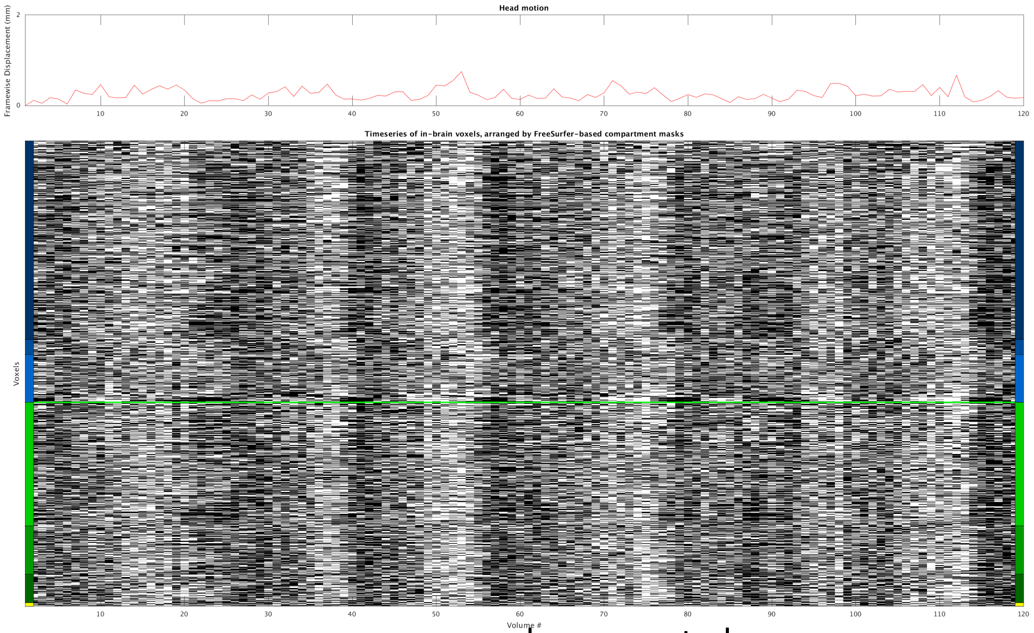

processed-uncorrected

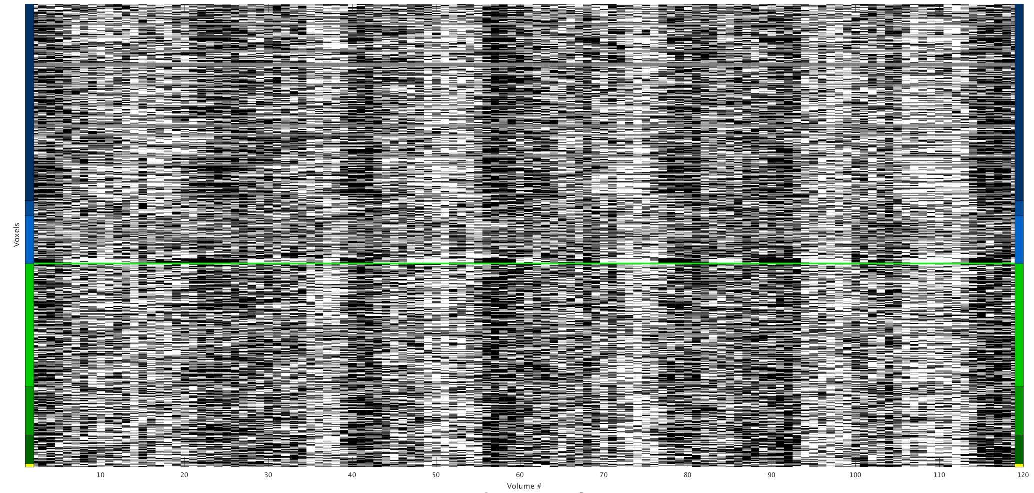

ICA-AROMA

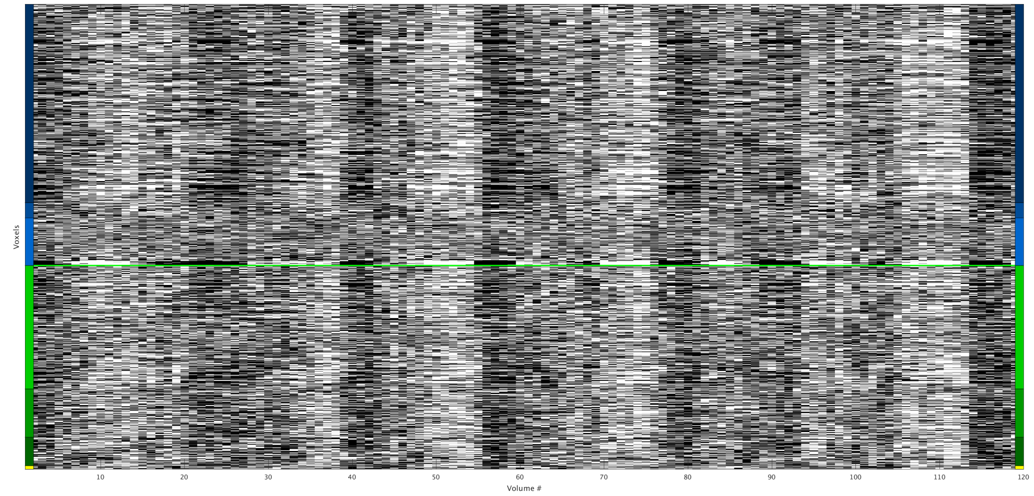

patient trained FIX

15

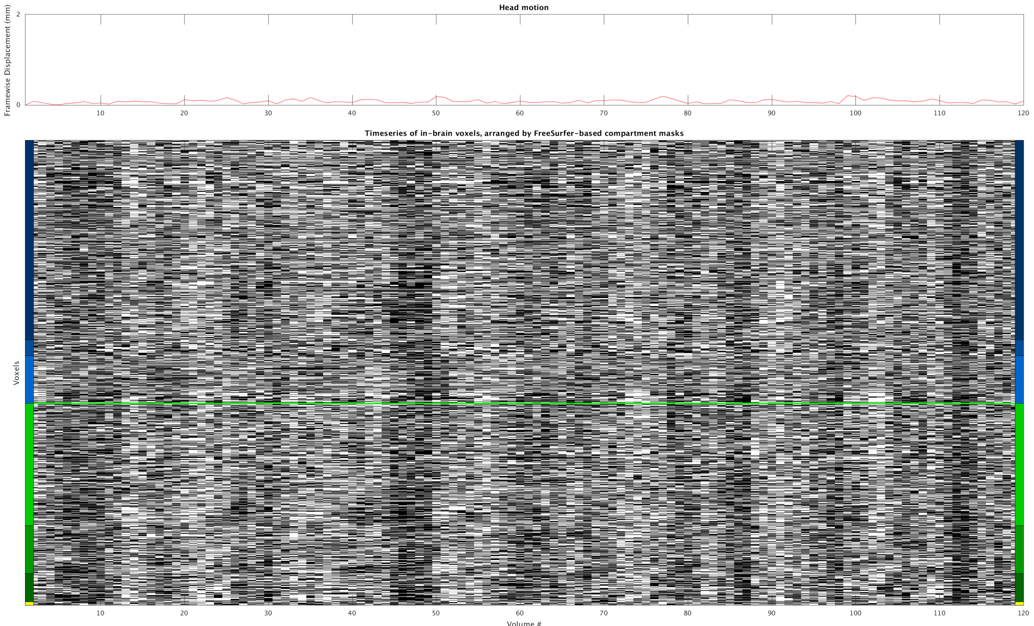

processed-uncorrected

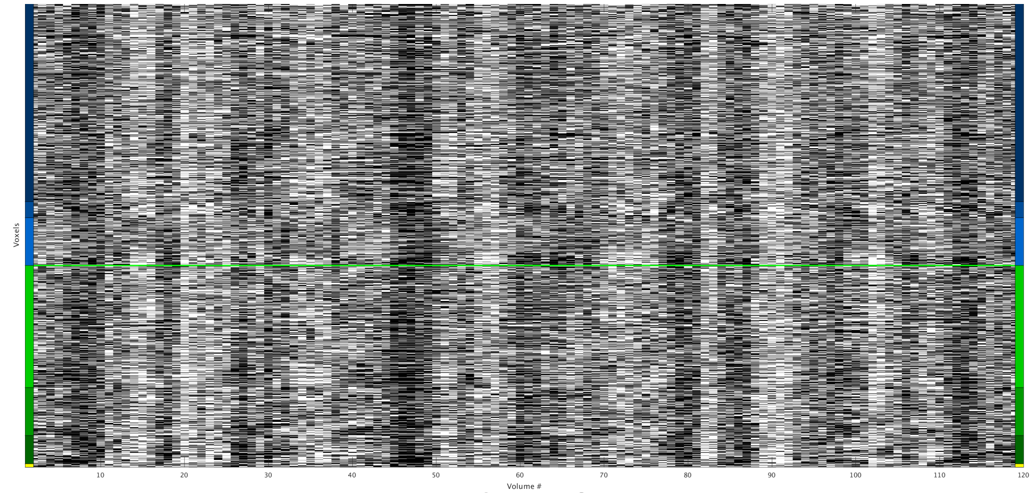

ICA-AROMA

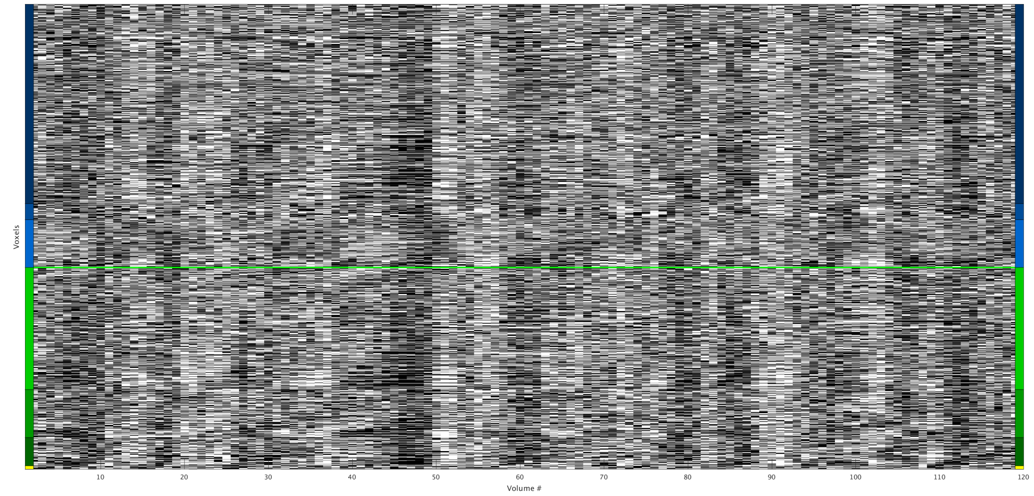

patient trained FIX

16

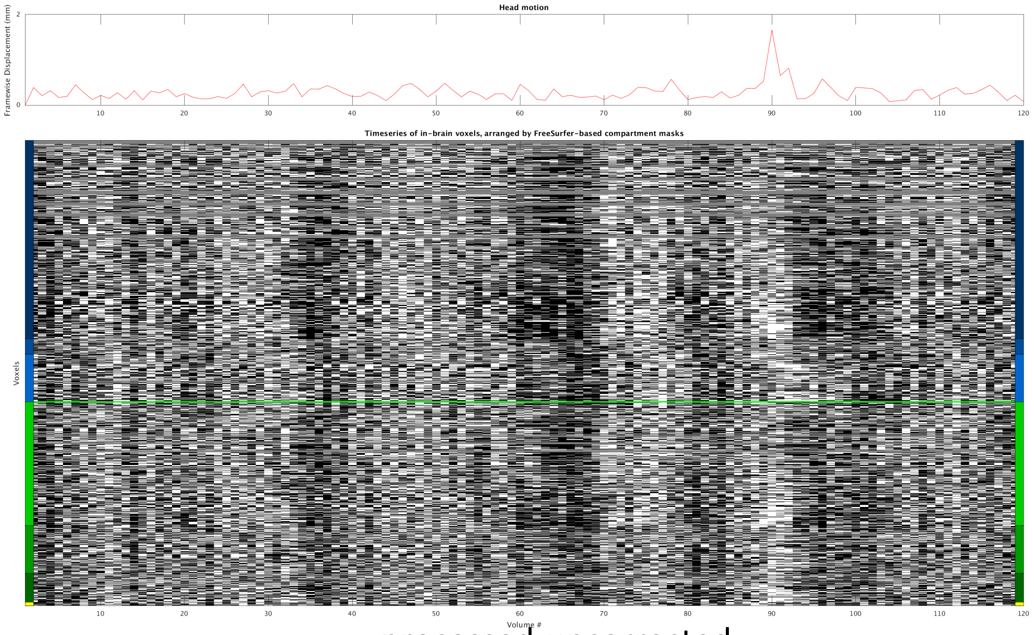

processed-uncorrected

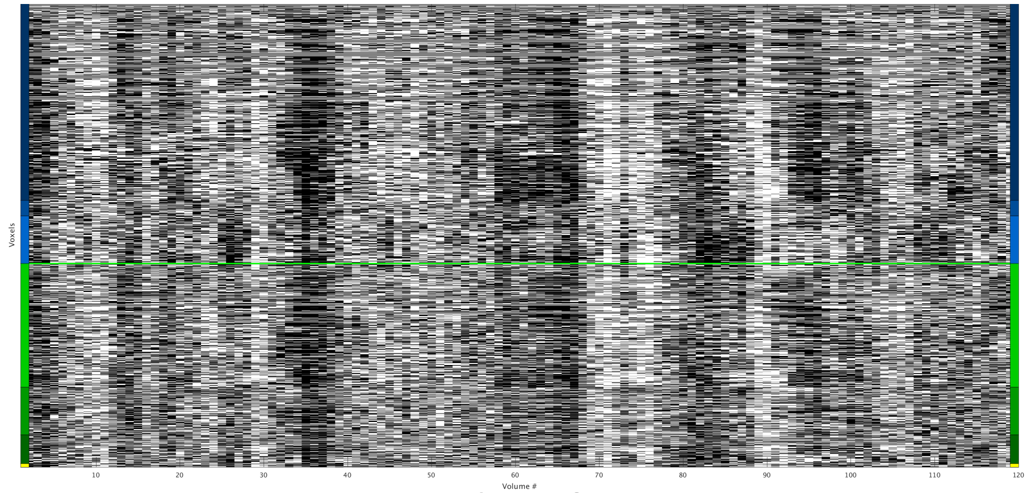

ICA-AROMA

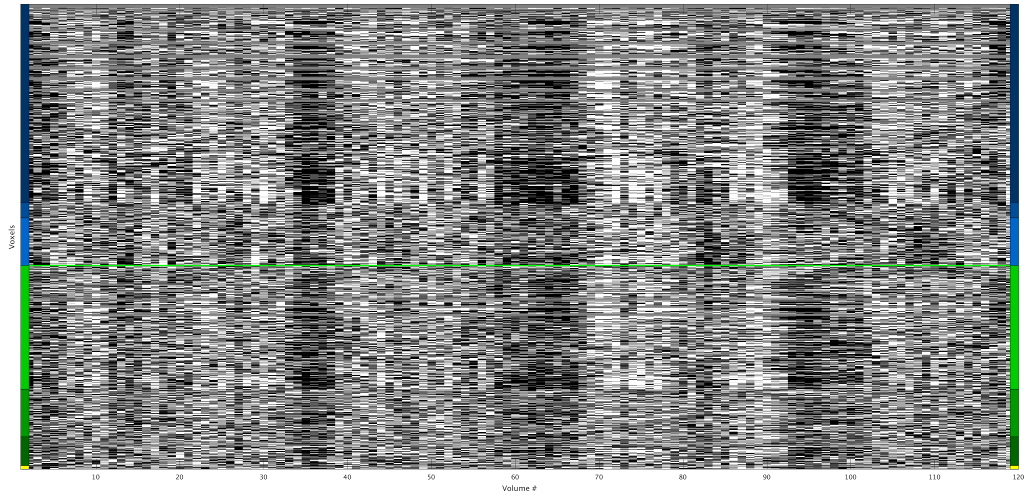

patient trained FIX

17

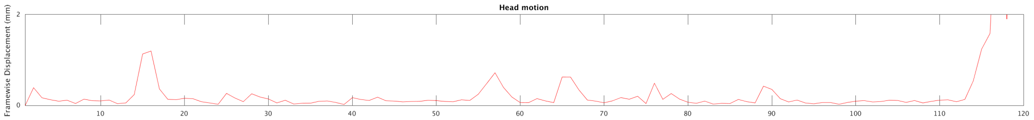

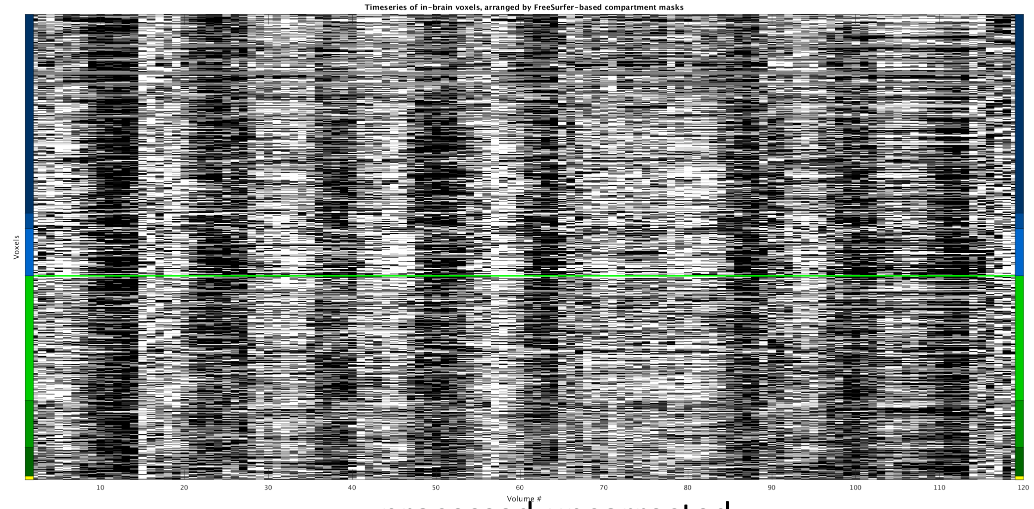

processed-uncorrected

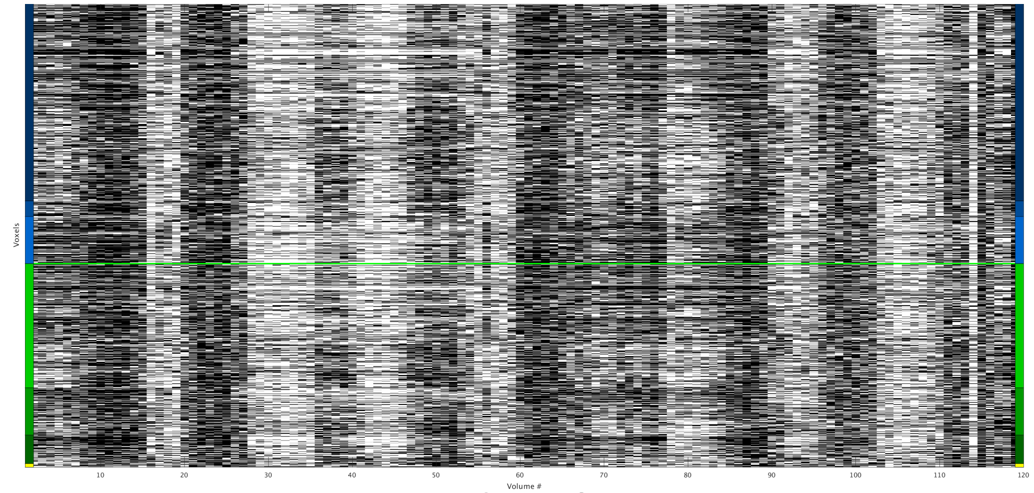

ICA-AROMA

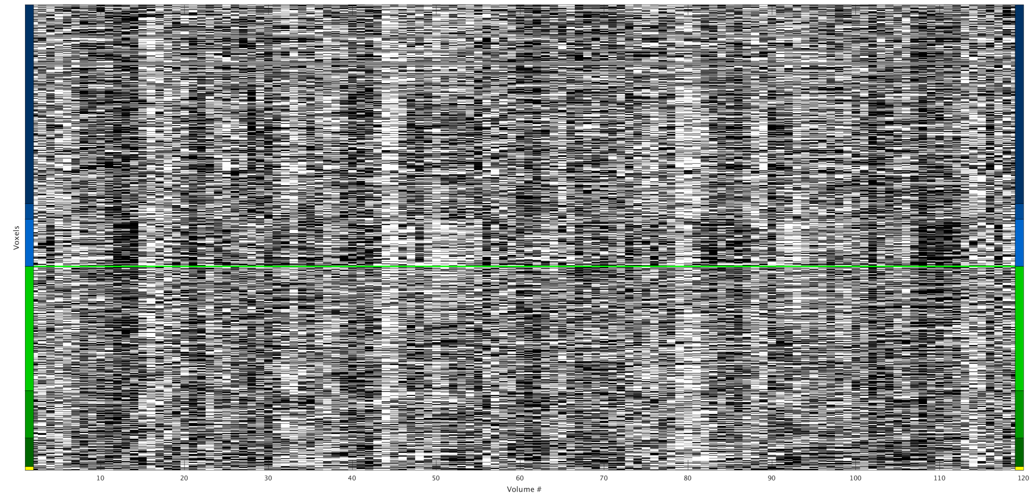

patient trained FIX

18

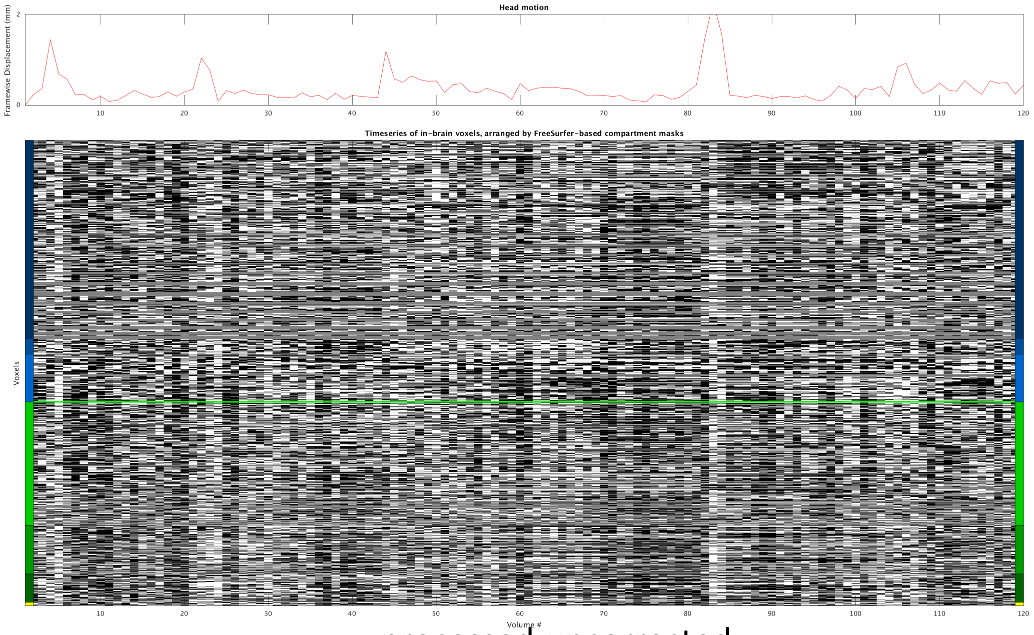

processed-uncorrected

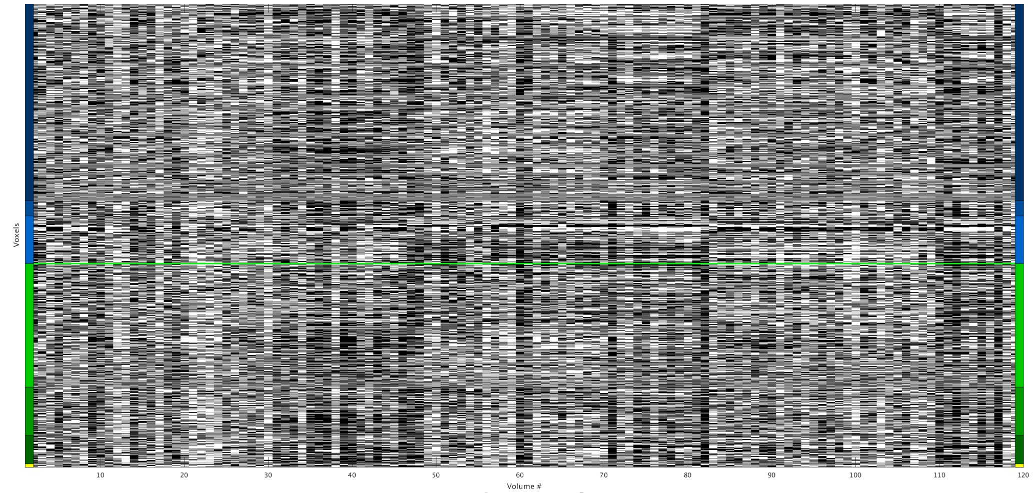

ICA-AROMA

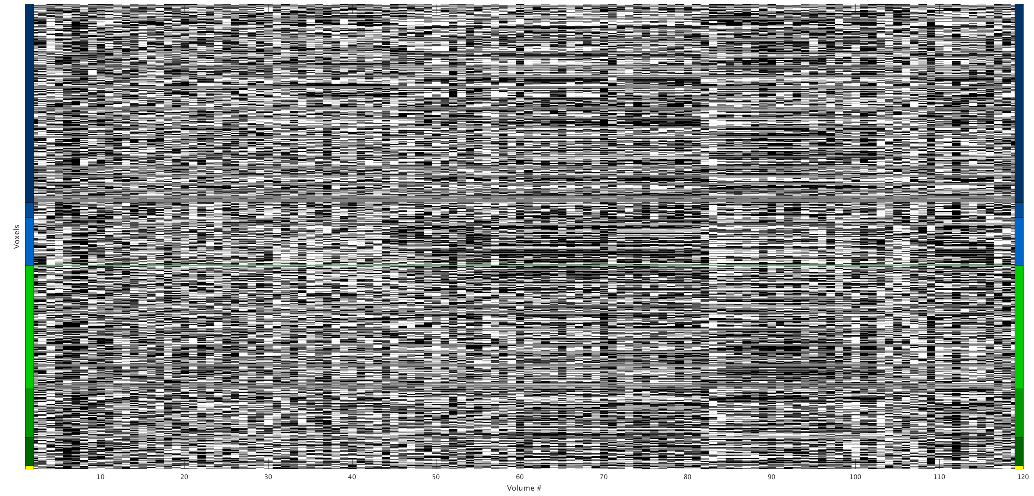

patient trained FIX

19

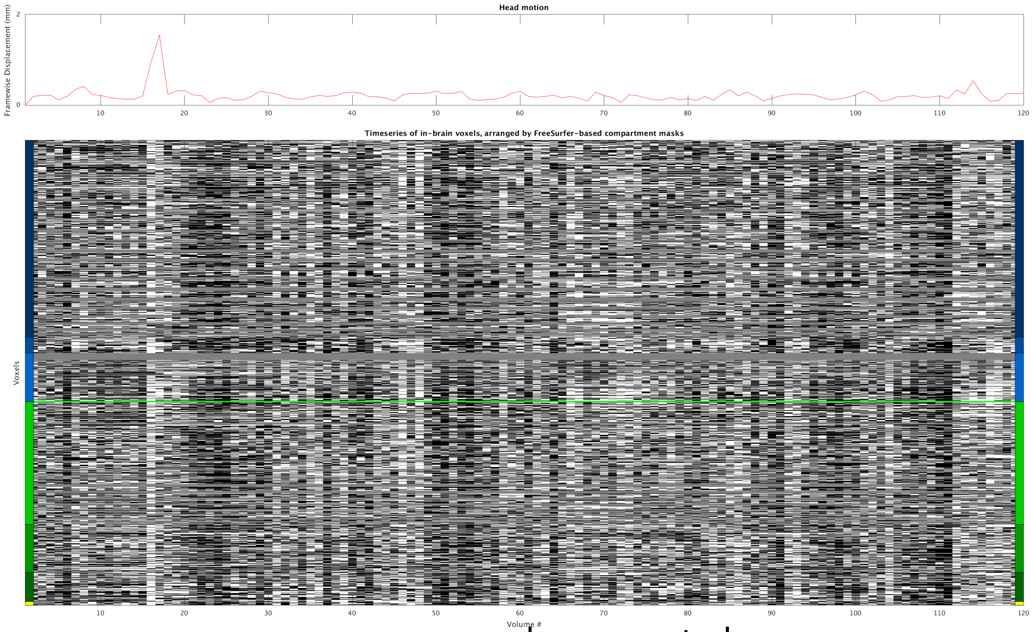

processed-uncorrected

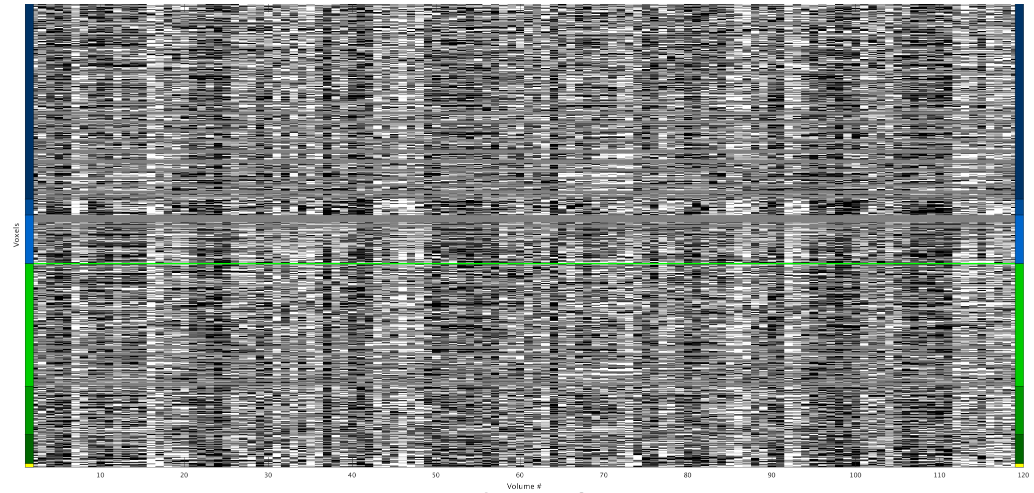

ICA-AROMA

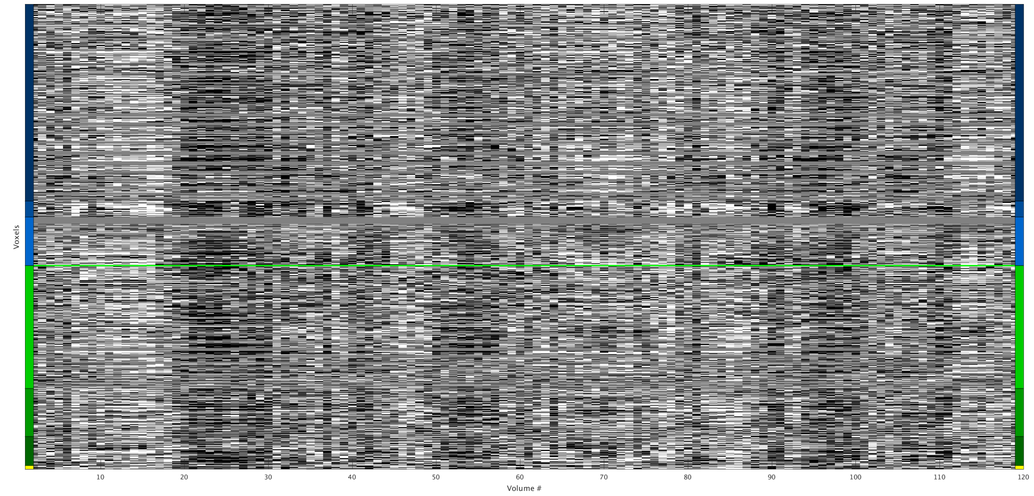

patient trained FIX

20

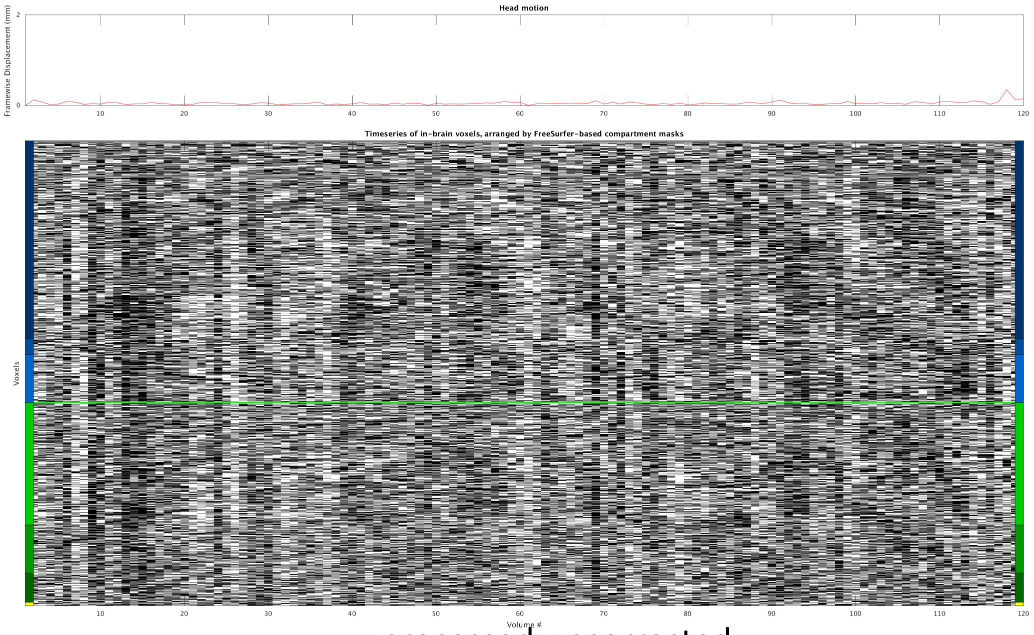

processed-uncorrected

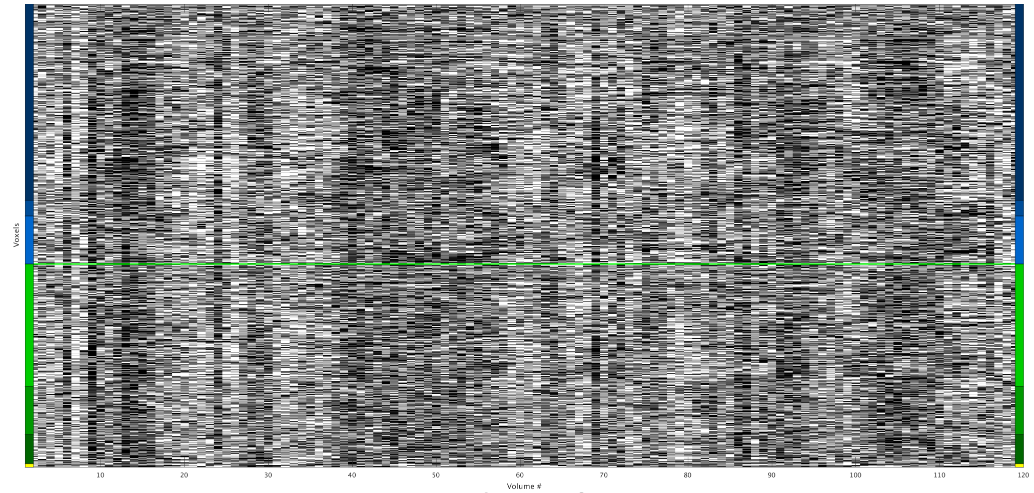

ICA-AROMA

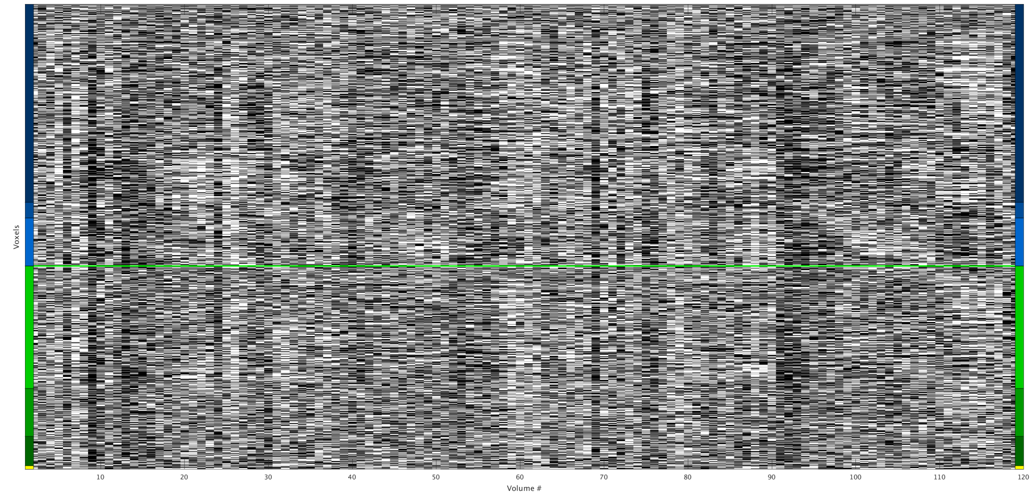

patient trained FIX
